# A novel behavioural INTErvention to REduce Sitting Time in older adults undergoing orthopaedic surgery (INTEREST): protocol for a randomised controlled feasibility study

**DOI:** 10.1186/s40814-019-0437-2

**Published:** 2019-04-06

**Authors:** Justin Avery Aunger, Colin James Greaves, Edward T. Davis, Carolyn Anne Greig

**Affiliations:** 1 School of Sport, Exercise, and Rehabilitation Sciences, University of Birmingham, Birmingham, B15 2TT UK; 2 Royal Orthopaedic Hospital NHS Foundation Trust, Bristol Road South, Northfield, Birmingham, B31 2AP UK

**Keywords:** Osteoarthritis, Sitting, Elderly, Older adults, Sedentary behaviour, Arthroplasty, Prehabilitation, Intervention, Feasibility study, Behaviour modification

## Abstract

**Background:**

Osteoarthritis is a highly prevalent condition in older adults, that causes many sufferers to require a hip or knee replacement in order to improve their quality of life and reduce pain. Individuals waiting for hip or knee replacements are often highly sedentary; thus, it is pertinent to assess whether reducing their sedentariness prior to surgery may aid in improving post-operative outcomes.

**Methods/design:**

The study will be a randomised controlled feasibility trial design, with 2:1 randomisation into an intervention and usual care group respectively. A target of 45 patients aged 60 years or older waiting for elective hip and knee replacements will be recruited from Russells Hall Hospital, Dudley, UK, approximately 8–10 weeks before surgery. The intervention, informed by Self-Determination Theory (SDT), will be composed of multiple behaviour change techniques, namely, motivational interviewing, feedback on current objectively measured sedentary behaviour and activity, goal-setting, environmental modification, self-monitoring, and social support. Assessments will occur at baseline, 1 week pre-surgery, and 6 weeks post-surgery. The primary outcome will be the feasibility of intervention delivery and of the trial procedures, assessed quantitatively based on rates of recruitment, retention, measures-completion, and intervention fidelity assessment, and with mixed-methods assessment of acceptability, practicality, adaption, satisfaction, and safety. Exploratory outcomes will include physical function, cardiometabolic biomarkers, measurement of SDT constructs, and both objective and subjective measurement of physical activity and sedentariness. The study will last up to 18 weeks per participant. No formal between-group comparisons are planned, but the variance in within-group changes and differences between groups in outcome measures will be explored and reported with 95% confidence intervals.

**Discussion:**

This is the first study assessing the feasibility of an intervention to reduce sedentary behaviour in older adults with mobility limitations, and the first to assess whether such a reduction could work in a prehabilitative context prior to surgery. The results of this study will help inform the design of a definitive randomised controlled trial.

**Trial registration:**

This trial is registered on Clinicaltrials.gov. Registration number: NCT03740412. Date of registration: 13/11/2018.

**Electronic supplementary material:**

The online version of this article (10.1186/s40814-019-0437-2) contains supplementary material, which is available to authorized users.

## Background

Sedentary behaviour, defined as “any waking behaviour characterized by an energy expenditure of ≤ 1.5 metabolic equivalent of tasks (METs) while in a sitting or reclining posture”, is associated with negative health outcomes [1]. In older adults, evidence suggests there is an association between sedentary behaviour and physical function, as well as cardiovascular disease, type II diabetes risk, and overall mortality [2, 3]. One study found that each additional 1-h period of sitting time per day is associated with a clinically significant reduction in physical function of 0.55 points of the Short Physical Performance Battery (SPPB) score [4, 5]. This is of particular concern, as studies using an objective measurement of sedentary behaviour have found that older adults (≥ 60 years old) spend an average of 8.5 h per day sitting [6].

Given the high degree of sedentariness in older adults, in combination with the mounting evidence of the associated health risks, several interventions aiming to reduce sitting time in older adults have been reported [11]. These include interventions to displace sedentary time to light physical activity such as walking, or simply other standing activities [7–12]. However, existing interventions have suffered several shortfalls. They are often feasibility trials with small sample sizes, and can lack objective measurement of sedentary behaviour using an inclinometer or accelerometer. Often, they have not assessed changes in blood biomarkers or physical function post-intervention, nor have they included follow-ups to assess the duration of behaviour change conferred [11]. Very few such trials have adequately reported the theoretical basis or provided a logic model underpinning their behaviour change intervention. Additionally, they have all included healthy older adult populations without significant comorbidity [11].

Older adults often have morbidities which can lead to even greater sitting time due to pain or other factors [13]. One such condition is osteoarthritis, which is extremely prevalent in older adults. In the UK, 18.2% of adults ≥ 45 years have osteoarthritis of the knee, and 10.9% have osteoarthritis of the hip [14]. Osteoarthritis causes chronic pain and further predisposes patients to be more sedentary. According to an analysis of objective sedentary behaviour data, individuals with mobility limitations have more sedentary time, less active time, and longer sedentary bouts compared with healthy controls [15]. Likewise, a recent review has identified that despite a reduction in pain after total hip or knee replacements, older adults do not return to being as active as their healthy counterparts [16].

Yet, no intervention to date has attempted to reduce the sedentary time in older adults with mobility limitations, despite the benefits it may bring to this at-risk population, and reducing sedentary behaviour in a prehabilitative context has not been explored. However, other studies to increase physical activity prior to arthroplasty have been found to be effective at improving physical function. A prior intervention utilising resistance, flexibility, and step training prior to knee arthroplasty in adults (*n* = 26) with a mean age of 64.1 has been found to be effective at reducing pain and improving sit-to-stand function [17]. Similarly, a study investigating the efficacy of a personalised physiotherapy programme in severely disabled adults (*n* = 28) with a mean age of 66.5 prior to knee or hip arthroplasty found significant improvement in timed up-and-go, self-paced walk, and timed stair tests after prehabilitation [18]. In terms of sedentary behaviour interventions, a previous study in older adults (*n* = 38) 60 years and over has found it possible to improve SPPB score by 0.5 points, a clinically meaningful increase, after a 12-week sedentary behaviour reduction intervention [19]. These data suggest that it may be possible to improve physical function simply by reducing sedentary behaviour. This may have potential as a method of prehabilitation.

### Aims

This study will assess the feasibility of delivering a novel intervention to reduce sedentary behaviour in a population of adults ≥ 60 years awaiting hip and knee arthroplasties in comparison with usual care. It will also assess the feasibility and acceptability of conducting the procedures required to conduct a full-scale RCT, by incorporating measurement of physical function, blood markers, objective measurement of sedentary behaviour, and physical activity, with both pre- and post-operative measurements.

The *primary* aim is to assess the study feasibility quantitatively using statistics related to the delivery of the study (uptake rate, recruitment rate, intervention adherence, delayed surgery, early surgery, cancelled surgery, retention rates, duration of intervention, session attendance); the *secondary* aim is to assess the feasibility outcomes of acceptability, practicality, satisfaction, and safety of the study in both arms using mixed methods; and the *exploratory* aim is to assess which of the efficacy measures (i.e. SPPB, cardiometabolic biomarkers, objectively measured sedentary time) are most promising for the design of a future clinical trial.

## Methods

This article has been written following both SPIRIT and TiDIER guidelines [20, 21].

### Study design

This is a single-site, parallel group, pragmatic randomised controlled feasibility trial design using 2:1 allocation into an intervention and usual care group, respectively.

### Study setting

This single-site study will be conducted at Russells Hall Hospital, Dudley, UK. Study procedures and intervention/assessment visits will take place either at participants’ own homes, at the hospital, or at the University of Birmingham, Birmingham, UK. This range of options is intended to decrease the burden to the participants.

### Patient identification

Patient lists obtained from orthopaedic clinicians by research nurses at Russells Hall Hospital will be screened for individuals fulfilling the study criteria. Patients will not be taking part in other interventions during participation in this study.

### Inclusion and exclusion criteria

Patients will be eligible if they are:

1. A man or woman aged ≥ 60 years.
2. Listed for elective hip or knee surgery.
3. Capable of providing informed consent as determined by their primary care team from medical records.
4. Regular access to a phone at pre-specified times.
5. Able to speak English.

They will be excluded if any of the following apply:

1. Neuromuscular impairments that preclude participating in physical activity, visual, hearing, or moderate/severe cognitive impairments as indicated by medical notes prior to recruitment.
2. Significant comorbid disease that would pose a safety threat, affect blood measures significantly, or impair the ability to participate in physical activity.
3. Unwillingness or inability to comply with the intervention.

### Recruitment process

**Fig. 1:**
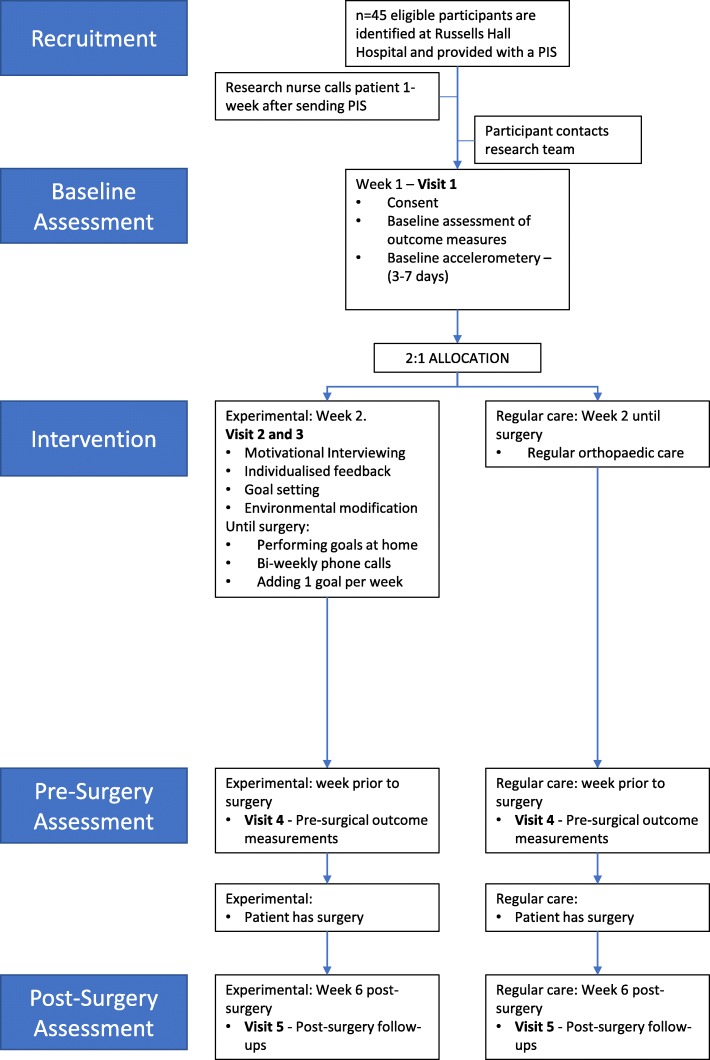
Study schema

The research nurses working at Russells Hall Hospital will screen patient lists to identify patients scheduled for hip or knee replacements and who meet inclusion and exclusion criteria. Research nurses will send participant information sheets (PIS) by post to eligible patients, and the patients will be asked to either return an enclosed contact agreement form or contact the research team by phone or email directly to indicate their interest in taking part (Fig. 1). Research nurses will also phone the participant 1 week after sending the PIS to ask whether they are interested in taking part. Wherever possible, patients will be enrolled in the study 8–10 weeks prior to their estimated surgery date.

### Sample size

A power calculation informed us that a sample size of 44 is sufficient to allow estimation of the retention rate of a future clinical trial with 95% confidence intervals of ± 11%, given an expected retention rate of 80%. For this reason, this study aims to recruit 45 patients, with 30 randomised to the intervention group and 15 to the usual care group. This sample size is also consistent with advice from clinicians about expected uptake rate and sample sizes used in prior feasibility studies to reduce sedentariness in older adults [11] This sample will be recruited over a 12-month period.

### Informed consent

The patient will provide written informed consent at the initial study visit.

### Randomisation and group allocation

Due to the nature of the intervention, it is not possible to blind participants or the data-collecting researcher to group allocation. Randomisation will be conducted using 2:1 permuted block randomisation by a third-party researcher, not affiliated to the project, who will retain allocations in confidence. This 2:1 allocation ratio was chosen to increase the number of people in the intervention group to allow for greater assessment of the feasibility of the intervention (Table 1). The primary researcher will be blinded to the allocation until the date of the visit in which the intervention must be delivered for each participant.

**Table 1:** Template for Intervention Description and Replication (TIDieR) checklist table [21]

Brief name	Pre-surgical sedentary behaviour reduction in orthopaedic patients
Why	Reducing sedentary behaviour prior to surgery has the potential to improve physical function and cardiometabolic health, leading to better post-surgical recovery.
What (materials)	As part of the intervention, a custom-made booklet will be used to aid in delivery of the education, goal setting, and environmental modification components, with space to record adherence to planned actions and environmental modifications.
What (procedures)	The intervention itself consists of a few key procedures: 1. Motivational interviewing to reduce ambivalence towards sedentary behaviour. 2. Education about sedentary behaviour and its negative health consequences. 3. Goal-setting and environmental modification. 4. Self-monitoring using a pedometer and recording of goal adherence. 5. Biweekly phone calls to maintain participant motivation and address issues.
Who provided	The intervention will be delivered solely by a trained researcher (JA), who has had training in motivational interviewing and who designed the intervention and materials. In this manner the intervention delivery will be very consistent.
How	Intervention content will be provided in a face-to-face and one-to-one context.
Where	The intervention and assessment sessions will occur mostly in participants’ own homes, but some sessions can occur at Russells Hall Hospital or at the University of Birmingham.
When and how much	The intervention can be delivered in 120 min in a single session followed by three 10-min biweekly phone calls. However, the study also includes 3 assessment points at baseline, 1 week pre-surgery, and 6 weeks post-surgery. Participants are expected to join the study 8–10 weeks before their surgery, and total study duration may last up to 18 weeks per participant.
Tailoring	As the goals and environmental modifications are the active behaviour change component of the intervention, each participant will have a wholly bespoke experience tailored to their individual capacity for physical function and their lifestyle.
Modifications	Goals and environmental modifications may be altered at any point by the participant if they are finding it hard to achieve, e.g. they could lower their step count target, or alter the frequency of another goal’s occurrence. The researcher will stay informed of these changes using the phone calls.
How well planned (fidelity)	A subset of the motivational interviews will be recorded and assessed for quality against a checklist of motivational interviewing techniques and content. An additional subset of these interviews will be recorded using field notes. A subset of phone calls will be checked for content delivery by the researcher against a purpose-built checklist. Action plans will be checked by an independent reviewer for adequacy, adherence to SMART principles, and appropriateness to the participant.

### Behavioural intervention

#### Theoretical framework

**Fig. 2:**
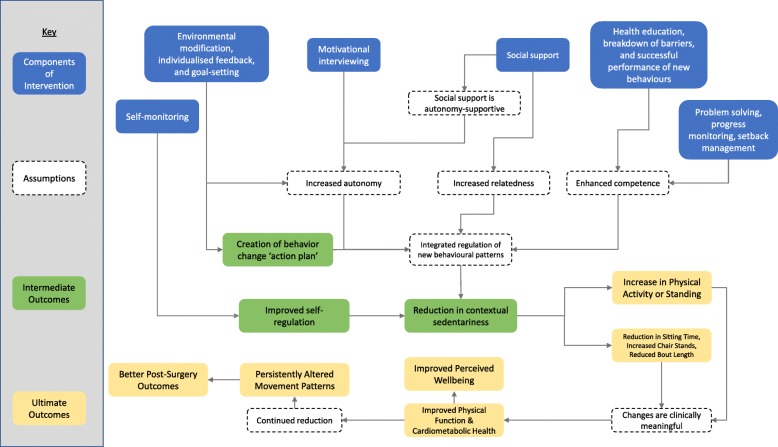
Logic model and integration of SDT into the INTEREST study’s design

This intervention was developed at the University of Birmingham by the study team and uses Self-Determination Theory (SDT) as a framework to help support behaviour change [22]. SDT is primarily a theory of motivation, and it posits that individuals have intrinsic desires to maximise achievement towards their long-term goals [23]. SDT is based on the premise that individuals have three fundamental needs: autonomy, competence, and relatedness, that must be satisfied for optimal motivation [24]. Consideration for supporting these basic psychological needs was used throughout the intervention design process (e.g. during selecting of behavioural change techniques (BCTs) and in specifying the intended consultation style) and the Basic Psychological Needs Scale was incorporated as an outcome measure to assess changes in these constructs over the course of the intervention [25, 26]. A sub-theory of SDT, called organismic integration theory, states that by supporting Basic Psychological Needs, extrinsically regulated behaviours (i.e. new “goals” revolving around sitting less and being more active) can move from a state of external regulation to integrated regulation [27]. In integrated regulation, these new behaviours align with the individual’s personal needs, and thus, the frequency of these behaviours will be maximised (Fig. 2).

#### Intervention materials

**Fig. 3:**
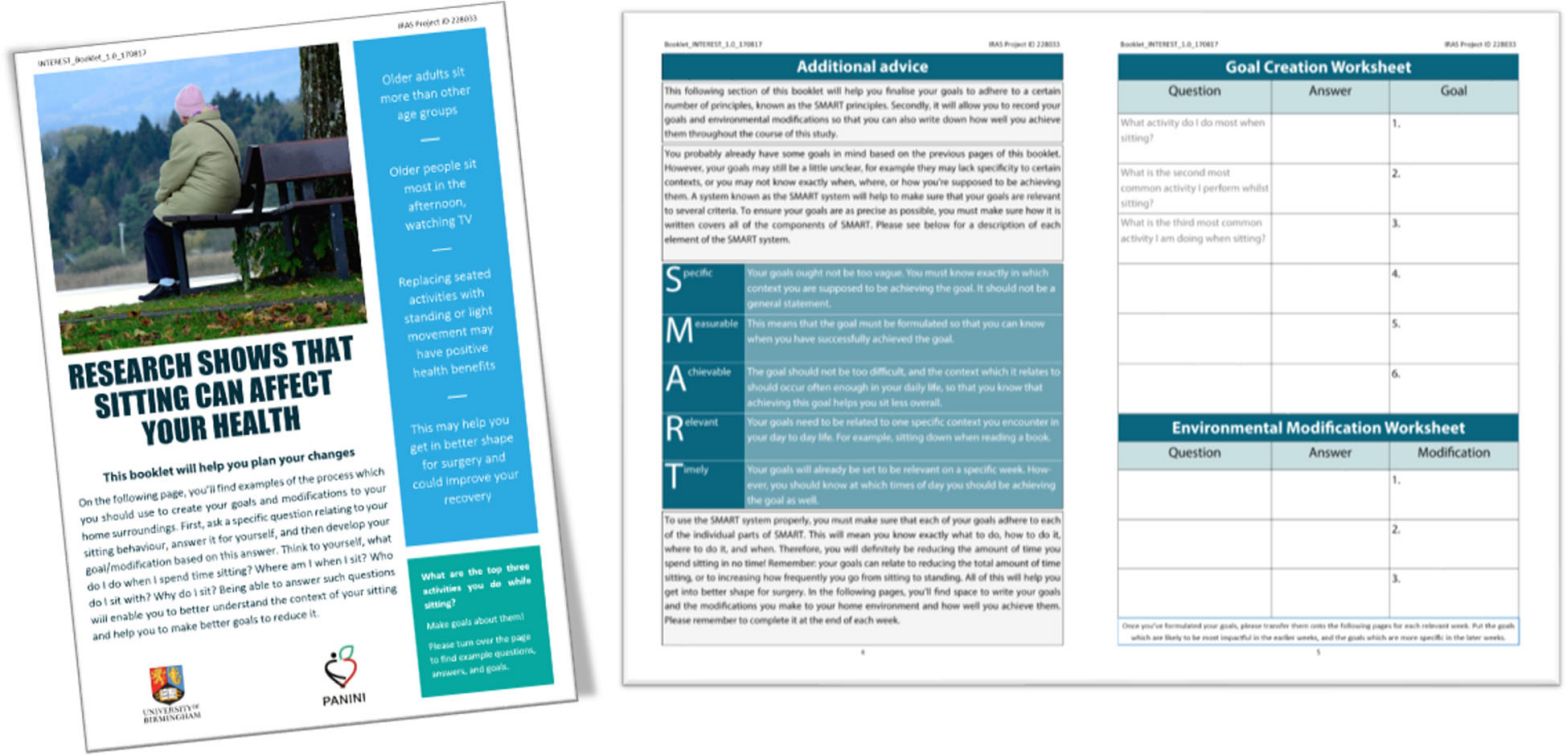
Sedentary behaviour booklet developed for INTEREST

A sedentary behaviour booklet was developed and used in this intervention (see Additional file 1; Fig. 3). This booklet provides some brief education about the effect of sitting on health, walks participants through the process of making an action plan in collaboration with the researcher, provides space for them to write the plan, and offers space for recording goal adherence. Additionally, a Yamax SW200 Digi-Walker pedometer was provided to participants to allow them to track their daily step counts.

#### Intervention components

A previous review of studies incorporating interventions to reduce sedentary time in adults aged 18 years and older found that those which included more BCTs were more effective than those that involved fewer [28]. By reviewing the literature, we were also able to identify which behaviour change techniques were used in existing interventions in older adults, which was used to inform the present intervention [11]. The full rationale for these components is provided in Additional file 2.

The intervention is built upon the following main techniques:

- *Education:* Written information will be provided in a custom-designed “sedentary behaviour booklet” (Additional file 1) that summarises the latest research about sedentariness in older adults in a simple way. Verbal information will also be provided where appropriate during conversations with the participants.
- *Motivational interviewing*: The participant will take part in a motivational interviewing session of approximately 30 min during visit 2, designed to reduce ambivalence around the participant’s sitting and physical activity, to identify enablers, to break down barriers, and to explore and enhance the participant’s ability to change [29]. Motivational interviewing has been found to integrate well with SDT, as MI aims to support patient autonomy and the other basic psychological needs [30, 31]. These sessions will be delivered by a researcher trained in motivational interviewing (JA).
- *Individualised feedback:* In visit 1, participants will be given an accelerometer to wear for 3–7 days (activPAL, PAL Technologies, Glasgow, UK). Their summary baseline data will be shown to participants in the form of an easy to understand report generated by the bespoke software in visit 3. This will help inform the participant and the researcher about the participant's relative sedentariness and activity and to allow targeting of specific times of day with their goals.
- *Individualised, incremental goal setting*: In visit 3, participants will formulate six goals with the aid of the researcher, in a collaborative process that encourages them to generate their own ideas. These plans will be set using SMART criteria for each goal (i.e. specific, measurable, achievable, relevant, and timely). For example, participants might plan to “walk to the kitchen and back during every TV break” or “do five consecutive chair rises after every meal each day”. These goals are designed to reduce the length of sedentary bouts, reduce total sedentary time, and increase sit-to-stand transitions. Participants will be encouraged to add one goal per week, so in week 1, they are working on one goal, in week 3, they are working towards three goals, etc., up to 6 weeks and six goals. Goals will be recorded in the sedentary behaviour booklet.
- *Environmental modification:* Participants will make three modifications to their environments in a way that is supportive of a less sedentary lifestyle and will record these in the booklet. These could include something as simple as keeping the TV remote next to the TV so that they must get up to change the channel.
- *Self-monitoring:* Participants will be provided with a pedometer, with which they can track their step counts, and a space within the booklet where they can record their adherence to the action plan and document any problems they encountered.
- *Social support:* In some cases, it will be possible to incorporate spousal or familial support into the goals and environmental modifications made by the participants to help with adherence and encouragement. In addition to this, three biweekly phone calls with the researcher will use motivational interviewing techniques, where participants will be asked about progress on their goals and whether they have made any modifications to them, etc. This will aid the maintenance of participant motivation and adherence.

#### Intervention structure and delivery

Participants will receive up to five visits (Table 2). The usual care group will attend only three visits, namely, 1, 4, and 5, which are assessment-only visits. The intervention group, however, will also attend visits 2 and 3 (which may be combined if time permits). Visit 2 consists of the motivational interview, whereas visit 3 involves the goal-setting, environmental modification, scheduling of the phone calls, and provision of the pedometer. The intervention is designed to be non-burdensome to researcher and patient in order to make it realistically implementable in orthopaedic care, as the motivational interviewing, education, goal-setting, and environmental modification session can be delivered within a single 90–120-min session, with the addition of three 10-minute phone calls.

**Table 2:** Standard Protocol Items Recommendations for Interventional Trials (SPIRIT) diagram to show the participant schedule, including enrolment, allocation, interventions, visits, and assessments [20]

	Study period										
	Recruitment	Baseline	Allocation	Post-allocation							Close-out
Visit	Pre-enrolment	Visit 1	Allocation between visit 1 and 2	Visit 2	Visit 3	PC 1	PC 2	PC 3	Visit 4	Visit 5	Post-study
Timepoint (weeks)	1	2	3	3	3	5	7	9	1 week prior to surgery	6 weeks post-surgery	
Study member	Research nurse	R	Third party	R	R	R	R	R	R	R	R
Enrolment											
Eligibility screening	X										
Informed Consent		X									
Allocation			X								
Study groups											
Sedentary behaviour reduction	X	X	X	X	X	X	X	X	X	X	
Regular care	X	X	X						X	X	
Assessments											
Feasibility (study statistics)											X
Feasibility (interviews with research nurses)											X
Feasibility (questionnaire, acceptability, practicality, adaption, satisfaction, safety)									X	X	
Socio-demographic questions (age, gender, ethnicity, prior occupation, country of origin, educational level, pet ownership, marital status, living arrangements, alcohol frequency smoking frequency, medication info, medical history)		X									
activPAL measurements (sitting time, sit-to-stand transitions, no. of sedentary bouts ≥ 30 mins, avg. length of sedentary bouts, stepping time, standing time, steps per day)		X							X	X	
International Physical Activity Questionnaire–Short Form (IPAQ-SF) [39]		X							X		
Measure of older adults’ sedentary behaviour (MOST) [40]		X							X	X	
Quality of life (QoL) (EuroQoL 5D-5L, EuroQoL Visual Analogue Scale) [41]		X							X	X	
Oxford Hip and Knee Score(s) [42, 43]		X							X	X	
Basic Psychological Needs Scale [25, 26]		X							X	X	
Activities of daily living (ADL) (Katz-ADL) [37]		X							X		
Physical function–Short Physical Performance Battery (SPPB) [36]		X							X	X	
Short Form Mini Nutritional Assessment (SF-MNA) [37]		X									
Weight		X							X	X	
Height		X									
Body mass index		X							X	X	
Waist-to-hip ratio		X							X		
Albumin, high-density lipoprotein, low-density lipoprotein, cholesterol, triglycerides, vitamin D, cortisol, transferrin, HBA1c, CRP, full blood count		X							X		

*R* researcher, *PC* phone call, *CRP* C-reactive protein

Due to the variation in surgery scheduling and the difficulty in obtaining accurate surgery dates months in advance, not all participants may undergo the same duration of intervention. The intervention is intended to be of 6-weeks’ duration but can be extended or shortened where required. Individuals will receive visit 4 in the week leading up to their surgery and will receive visit 5 6-weeks post-operatively. Most individuals are expected to have surgery 4–10 weeks after visit 3. Participants are expected to participate in the study for a total of 15–18 weeks. The variability in intervention delivery and timings will be recorded to help inform the assessment of study feasibility.

#### Usual care

The usual care group will undergo three study visits: baseline (visit 1), pre-surgery (visit 4), and post-surgery (visit 5). Otherwise, they will have regular orthopaedic care in line with other non-study patients, but will be contacted by telephone every 2 weeks to follow up about the status of their ongoing care. However, the calls will not be pre-planned and will not entail any BCTs.

#### Intervention fidelity assessment

All goals and environmental modifications made during the study will be recorded and qualitatively coded after cessation of the study. Ratings of skill used in this study will utilise specifically-designed checklists based on the five-stage model of adult skill acquisition by Dreyfus et al., which has been used successfully in prior trials [32–34]. The fidelity tool is available in Additional file 3. Ratings of fidelity will be recorded digitally on forms specific to each intervention session and phone call and will be quantitatively analysed after cessation of the study.

Skills assessed will include motivational interviewing, problem-solving, progress monitoring, setback managing, and action planning. These will be assessed on a per-session basis where relevant. Action plans will also be reviewed for content, quality (adherence to SMART principles), and suitability for improving patient physical function. The researcher will self-rate a subset of action planning sessions for proper utilisation of all the relevant skills (e.g. whether patient autonomy was supported). A subset of the motivational interviews will be audio-recorded and checked for quality of delivery by an expert member of the study team (CG) using the same checklist. A further subset of the motivational interviews will be commented on by the researcher using field notes and subsequently qualitatively analysed. A subset of the phone calls will be immediately self-rated by the deliverer for the usage of motivational interviewing, problem solving, progress monitoring, and setback management skills.

### Study assessments

#### Primary outcome assessment

##### Feasibility (study statistics)

Feasibility will be assessed primarily using the following study statistics:

- Study uptake rate—percentage of participants whom receive a PIS who subsequently enrol in the study (%).
- Recruitment rates—average number of participants recruited per month.
- Intervention adherence—average self-reported goal adherence (scale 1–5) per week (recorded in sedentary behaviour booklet).
- Percentage of participants whose surgery occurs eight or more weeks after visit 3 (%).
- Percentage of participants whose surgery is scheduled four or fewer weeks after visit 3 (%).
- Percentage of participants with indefinitely delayed or cancelled surgery—proportion of participants who will not have surgery within the lifetime of the study (%).
- Retention rates—percentage of participants who remain in the study (i.e. provide measures) and do not drop-out at each follow-up timepoint (%).
- Average duration of intervention—average number of weeks of participation in the intervention prior to surgery.
- Session attendance—number of intervention sessions attended and the associated total contact time.

#### Secondary outcome assessment

##### Feasibility questionnaire

Feasibility will be assessed secondarily by the use of bespoke questionnaires that comprise both open and closed questions: one for the usual care and one for the intervention group. Each question is designed to target an aspect of feasibility based on guidance by Bowen et al. [35]. The questionnaires assess acceptability, practicality, adaption, satisfaction/feedback, and safety/risk for the participant. These files are available as Additional files 4 (usual care) and 5 (intervention).

##### Qualitative interviews with healthcare staff

One semi-structured qualitative interview will be conducted after the cessation of recruitment with each research nurse who had a significant role in recruitment for the study. These will assess how feasible the recruitment process was from their perspective and provide valuable insight into whether improvements could be made to recruitment and study processes in a future definitive trial. A topic guide is available in Additional file 6.

#### Sociodemographic information

Data will be collected regarding participant age, gender, country of origin, language used at home, marital status, ethnicity, educational qualifications, years of school, main occupation, living arrangements, housing, pet ownership, alcohol drinking status and frequency, smoking status and frequency, and former smoking and drinking behaviour. Data regarding current medications and medical conditions will also be collected.

#### Exploratory outcomes

To assess the feasibility of collecting data to inform the efficacy of the intervention in this population, the following measures will be performed:

1. Physiological measurements: weight (kg), height (cm), body mass index (kg/m^2^), hip circumference (cm), waist circumference (cm), and waist-to-hip ratio (cm).
2. Objective assessment of sedentary behaviour and physical activity using activPAL3 inclinometers: daily sitting time, mean daily sit-to-stand transitions, daily number of sedentary bouts ≥ 30 mins, avg. length of sedentary bouts, stepping time (mean per day), standing time (mean per day), and mean steps per day.
3. Physical function: The Short Physical Performance Battery will be used. This gives a score of 0–12 across the domains of balance (4 points), 4-m walk test (4 points), and time to perform 5 chair rises without using the arms (4 points) [36].
4. Activities of daily living: Katz Activities of Daily Living Scale (0–6 score), based on a score for how independent participants are in bathing, dressing, toileting, transferring from bed to chair, continence, and feeding [37].
5. Nutritional status: Short Form Mini Nutritional Assessment, assesses the risk of malnutrition (score 0–14) [38].
6. Subjective assessment of physical activity: International Physical Activity Questionnaire (Short Form) [39]. Provides self-reported data regarding minutes of vigorous physical activity (h/week), moderate physical activity (h/week), walking (h/week), and sitting (h/day).
7. Subjective assessment of sitting: Measure of Older Adults’ Sedentary Behaviour [40]. Assesses self-reported sitting hours per week in multiple behaviours: watching television/video, time on computer/internet, reading, socialising with friends/family, driving or riding in a vehicle, hobbies, and other activities (will be reported in total hours/day, and hours/week for each sub-domain).
8. Quality of life: EuroQol 5D-5L and EuroQoL Visual Analogue Scale [41]. The former consists of 1–5 scores across domains of mobility, self-care, usual activities, pain/discomfort, and anxiety/depression, and the latter consists of a 0–100 self-rating of overall health. The former will be reported for each sub-domain.
9. Impact of osteoarthritis: Oxford Knee Score and Oxford Hip Score [42, 43]. This provides a score from 0 to 48 across 12 questions with 5 options each, where a score of 0 is the most severe. The scale covers pain, impact on daily activities, and more. Overall score will be reported.
10. Basic psychological needs: Basic Psychological Needs Scale in General [23, 25]. This provides a score for autonomy, competence, and relatedness across 21 questions (7 each). Scores of 0–7 are output for each need.
11. Blood test–cardiometabolic biomarkers: albumin (g/L), high-density lipoprotein (mmol/L), low-density lipoprotein (mmol/L), cholesterol (mmol/L), triglycerides (mmol/L), vitamin D (ng/mL), cortisol (nmol/L), transferrin (g/L), HBA1c (nmol/mol), and C-reactive protein (mg/L). Blood will be collected via venepuncture at the site of the visit by a trained phlebotomist and taken immediately post-visitation for analysis at the University Hospitals Birmingham Clinical Laboratory Services. This blood is not stored.

The measurement timepoints are listed in Table 2, and the rationale for these measures can be read in Additional file 7. These data will inform the design of a definitive trial by helping to identify which outcomes are feasible, which demonstrate the greatest responsiveness to the intervention, and what sample size is needed to provide sufficient statistical power to detect changes in the primary outcome (calculated from standard deviations for changes in the outcome measures). Data will be retained and analysed on an intention-to-treat basis.

### Data analysis

Data from the quantitative assessment of feasibility will be presented using simple descriptive statistics (e.g. means, standard deviations, proportions, change scores, and confidence intervals).

Qualitative data collected to inform feasibility will be thematically analysed and coded using NVivo 12 software (QSR International). This includes interviews with healthcare staff, open-ended questions on the feasibility questionnaires, notes made during feasibility assessment, and notes made by researchers following motivational interviews. Additionally, participant goals will be coded into categories according to what type of behaviour they are targeting, the physical function status of the patient with whom they were created, and more, to create a framework of goals achievable by those with differing degrees of mobility. A combination of deductive and inductive coding will be used depending on the data source; for example, participant goals will be deductive, and open-ended questions inductive.

Exploratory analyses of outcome data will be conducted using IBM SPSS Statistics 25.0. These will comprise of ANCOVA analyses for the measures taken only at baseline and pre-surgery and 2 × 3 ANCOVAs for the data collected at all three timepoints to explore the variance in between-group differences and within-group change scores. We will not formally test for the significance of these differences but will report the mean differences (within and between groups) with 95% confidence intervals.

### Progression to a definitive trial

Progression to a definitive trial will be considered if the following metrics are met:

1. A minimum of 75% of patients have their surgeries within 10 days of the 4–8-week intervention window between visit 3 and visit 4.
2. Rate of uptake meets or exceeds 10%.
3. Participant retention rate exceeds 75% between baseline and pre-surgery visits.
4. Study satisfaction must be ≥ 4/5, and risk of harm should be < 2/5, as assessed by the feasibility questionnaire given to participants.
5. The frequency of adverse events does not call into question the safety of the trial as determined by the medical expert on the study (ETD).

The rationale for these criteria is as follows:

1. Participants need to have their surgery within a specific timeframe to keep the intervention length somewhat equivalent. This may be alleviated if a definitive trial was multisite.
2. Based on advice from clinicians, approximately 300 patients are likely to be eligible to be sent a PIS over the 12-month recruiting period. An uptake rate of 10% would be below the target of 45 but would inform us that a multisite strategy would be necessary for the definitive trial.
3. A high number of drop-outs would inform us that the intervention is not acceptable in its current form. The post-surgery visit (visit 5) is not used as a criterion here, as whether patient surgeries are cancelled or delayed is unlikely to be related to study procedures.
4. The study should be perceived as safe and acceptable by participants.
5. Safety should be of primary concern, as increased walking and standing could potentially increase the risk of falls in this population.

### Trial Steering Committee

Due to the low risk associated with this small-scale feasibility study, a formal Trial Steering Committee (TSC) has not been commissioned; however, the University of Birmingham Research Compliance Team will review the study’s progress and any adverse events at intervals agreed with the Chief Investigator (CI).

### Data monitoring

No Data Monitoring Committee will be commissioned for this study due to its small scale and low risk.

### Ethical considerations

A favourable opinion has been provided by a local NHS Research Ethics Committee (Solihull) in November 2017 (17/WM/0371), and the study will be conducted in concordance with Good Clinical Practice (GCP). Confirmation of capacity and capability was given by the Dudley Group NHS Foundation Trust on the 25 January 2018.

### Safety monitoring

All potentially serious adverse events, defined as any event that could be related to the study and that caused injury or hospitalisation, will be reported to the principal investigator and the study’s clinical orthopaedic consultant (ETD) within 24 h of the team being aware of its occurrence and will be reported to the sponsor within the same period. Additionally, any concerning blood results or evidence of risk of malnutrition will be reported to the principal investigator and the patient’s medical team.

### Auditing

The study team will allow monitoring of the study by the sponsor and other regulatory bodies.

### Dissemination policy

A full study report will be produced within 6 months of the end of the trial and, if published, will be provided in an open access format. Only the CI will publish any of the resulting data, within which acknowledgements will be given to the sponsor and funding body. Participants will be contacted once publications have been produced and provided with access to the manuscript(s).

## Discussion

To our knowledge, this is one of few behaviour change interventions to be delivered to individuals undergoing hip and knee replacement surgery, and the first sedentary behaviour intervention to be delivered to older patients with mobility limitations. A further novelty is that this study will assess the feasibility of both objectively-assessed intermediary mediators (i.e. sedentary behaviour, activity) and longer-term clinical outcomes (i.e. blood measures, physical function), which is a key step forward in sedentary behaviour intervention research in older adults.

Limitations of this study include the variable intervention length, which means that participants may acquire differing degrees of benefit from the intervention, and its single-site design, which means that it may not be applicable to other hospital settings. Also, a single-site design was chosen due to resource limitations and to minimise between-site effects on study outcomes; however, this may mean that feasibility data may not be as applicable to a future multisite trial. Additionally, some of the fidelity assessments are self-rated, which increases the likelihood of bias, and participants and researchers are not blinded to group allocation. Lastly, the 6-week follow-up may still be insufficient to assess full recovery post-surgery, and insufficient integration with the healthcare team meant that data regarding hospital admissions, surgery complications, etc., could not be collected. These limitations are important design considerations for a definitive trial.

The robust measurement of feasibility through the mixed-methods techniques outlined above and the assessment of fidelity at multiple delivery and implementation points will inform the design of a future definitive clinical trial.

### Trial status

The study opened to recruitment on the 29 January 2018 and is currently ongoing. Recruitment is expected to cease in January 2019 with the study completing in April 2019.

## Additional files

Additional file 1: INTEREST Sedentary Behaviour Booklet. Booklet used in the study in which the participant can learn about sedentary behaviour and about the action planning process. It also has space within for setting goals and recording intervention adherence. (PDF 3560 kb)

Additional file 2 Theoretical development of INTEREST. (DOCX 49 kb)

Additional file 3: INTEREST Fidelity Toolkit. Document detailing the fidelity assessment processes in the INTEREST study. (DOCX 48 kb)

Additional file 4: INTEREST Feasibility Questionnaire (control group). Feasibility questionnaire given to participants at visit 4 (pre-surgery) and visit 5 (post-surgery) to assess acceptability, adoption, practicality satisfaction, and safety of the study and to get additional feedback on study processes. (DOCX 22 kb)

Additional file 5: INTEREST Feasibility Questionnaire (intervention group). Feasibility questionnaire given to participants at visit 4 (pre-surgery) and visit 5 (post-surgery) to assess acceptability, adoption, practicality satisfaction, and safety of the study and to get additional feedback on study processes. (DOCX 27 kb)

Additional file 6: Topic guide for the interview with research nurses. Topic guide to provide information about what the interview regarding feasibility of recruitment with research nurses entails. (DOCX 16 kb)

Additional file 7: Rationale for exploratory outcomes in the INTEREST feasibility study. (DOCX 47 kb)

Additional file 8: Participant informed consent form for the INTEREST study. (DOCX 127 kb)

Additional file 9: Participant information sheet for the INTEREST study. (DOCX 371 kb)

## Abbreviations

ADL: Activities of daily living
ANCOVA: Analysis of covariance
BCT: Behaviour change techniques
CRP: C-reactive protein
GCP: Good Clinical Practice
IPAQ-SF: International Physical Activity Questionnaire–Short Form
MOST: Measure of Older Adults’ Sedentary Time
PC: Phone call
PIS: Participant information sheet
QoL: Quality of life
R: Researcher
RCT: Randomised controlled trial
SPPB: Short Physical Performance Battery
TSC: Trial Steering Committee
