# Supplementary material for: A novel behavioural INTErvention to REduce Sitting Time in older adults undergoing orthopaedic surgery (INTEREST): protocol for a randomised controlled feasibility study
Source: Pilot Feasibility Stud. 2019 Apr 6;5:54. doi: 10.1186/s40814-019-0437-2 (PMC6451782; doi:10.1186/s40814-019-0437-2)
Supplement: Supplementary file 1 — INTEREST Sedentary Behaviour Booklet. Booklet used in the study in which the participant can learn about sedentary behaviour and about the action planning process. It also has space within for setting goals and recording intervention adherence. (PDF 3560 kb) [file 40814_2019_437_MOESM1_ESM.pdf]

Environmental Modification 1

Write your modification here: ________________ Start date: ___/___/______	Comments:
Date of review: ___/___/______ I have achieved this (circle a number): 1 2 3 4 5 Not at all A little Very well	Comments:

Environmental Modification 2

Write your modification here: ________________ Start date: ___/___/______	Comments:
Date of review: ___/___/______ I have achieved this (circle a number): 1 2 3 4 5 Not at all A little Very well	Comments:

Environmental Modification 3

Write your modification here: ________________ Start date: ___/___/______	Comments:
Date of review: ___/___/______ I have achieved this (circle a number): 1 2 3 4 5 Not at all A little Very well	Comments:

REMINDER: In week 3 and 5 you must add one environmental modification. So, in week 3 you'll be recording how you achieved 2 modifications, and in week 5, how you achieved 3 modifications.

INTEREST_Booklet_1.0_170817 IRAS Project ID 228033

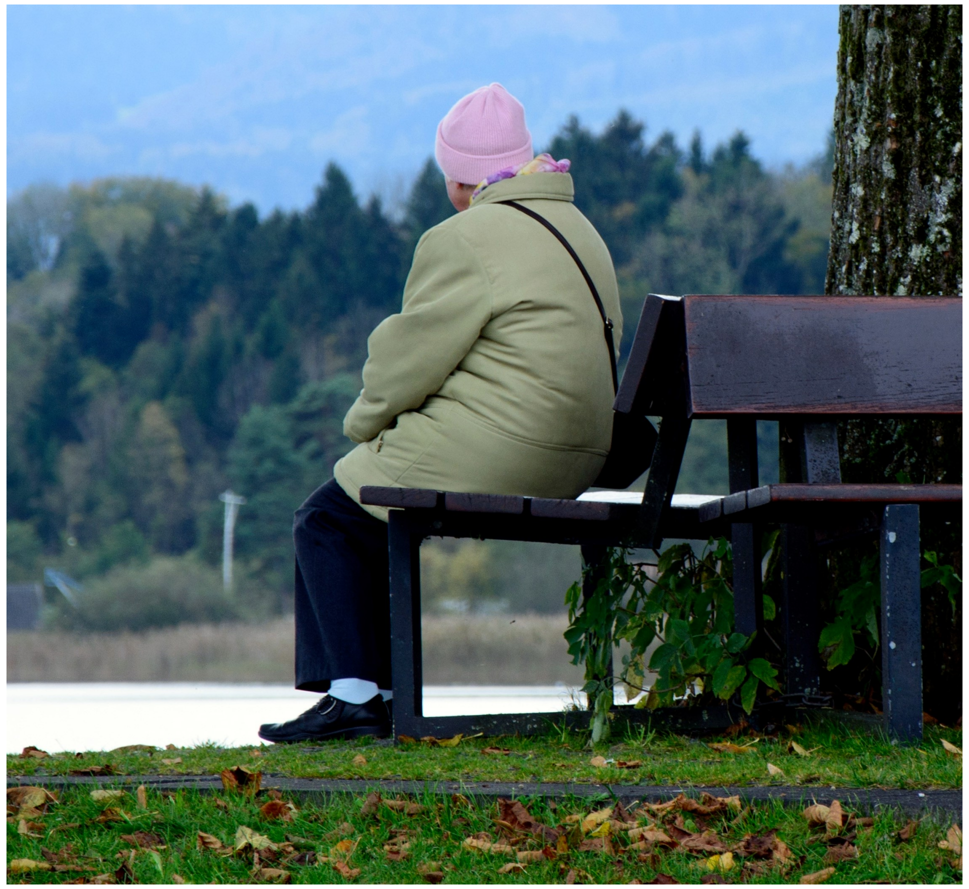

RESEARCH SHOWS THAT SITTING CAN AFFECT YOUR HEALTH

This booklet will help you plan your changes

On the following page, you'll find examples of the process which you should use to create your goals and modifications to your home surroundings. First, ask a specific question relating to your sitting behaviour, answer it for yourself, and then develop your goal/modification based on this answer. Think to yourself, what do I do when I spend time sitting? Where am I when I sit? Who do I sit with? Why do I sit? Being able to answer such questions will enable you to better understand the context of your sitting and help you to make better goals to reduce it.

UNIVERSITY OF BIRMINGHAM

PANINI

Older adults sit more than other age groups

Older people sit most in the afternoon, watching TV

Replacing seated activities with standing or light movement may have positive health benefits

This may help you get in better shape for surgery and could improve your recovery

What are the top three activities you do while sitting?

Make goals about them!

Please turn over the page to find example questions, answers, and goals.

Goal Creation Process

Question	Example Answer(s)	Example Goal(s)
Who am I usually with when I sit the most?	1. I am usually alone when I sit<br>2. I am usually with my family when I sit	1. Consider visiting friend or family once per day<br>2. Ask my family every day to encourage me to sit less
What activity do I do most when sitting?	Watching TV	Every time a new commercial break comes on or a programme ends,, walk to the kitchen and back
What is the second most common activity I perform whilst sitting?	Reading a book	Alternate reading chapters sitting and standing
What is the third most common activity I am doing when sitting?	Using the computer	Install a program that prompts me to take a walk every 20 minutes (e.g. http://monkeymatt.com/bigstretch/) - I can help you with this
Why do I sit in the dining room?	I sit in the dining room to eat	Stand up while eating breakfast every day
On which form of transport do I sit the most?	I sit the most in a bus	1. Get off one stop sooner on a bus trip and walk the rest of the way<br>2. Stand up while waiting for the bus
What is my main purpose for sitting?	For leisure	Do some housework or gardening or find a standing leisurely activity
In what time period of the day do I sit the most (e.g. morning, afternoon) based on my individualised feedback?	Afternoon	Add one more outdoor activity per day which takes place in the afternoon (e.g. gardening)
Where do I sit the most?	(More general) indoors	I must ensure I leave the house once every day

Worksheet: Week 4

**Write your goal here:**<br>____________________________________<br>**Start date:** ___/___/______	Comments:
Date of review: ___/___/______<br>I have achieved these goals (circle a number):<br>1 2 3 4 5<br>Not at all A little Very well	Comments:

Worksheet: Week 5

**Write your goal here:**<br>____________________________________<br>**Start date:** ___/___/______	Comments:
Date of review: ___/___/______<br>I have achieved these goals (circle a number):<br>1 2 3 4 5<br>Not at all A little Very well	Comments:

Worksheet: Week 6

**Write your goal here:**<br>____________________________________<br>**Start date:** ___/___/______	Comments:
Date of review: ___/___/______<br>I have achieved these goals (circle a number):<br>1 2 3 4 5<br>Not at all A little Very well	Comments:

REMINDER: For every week, you work towards the new goal for that week and all previous goals. For example, in week 2 you record achievement of goal 1 and goal 2, and in week 5 you record goals 1-5.

Worksheet: Week 1

**Write your goal here:** ______________________ **Start date:** ___/___/______	Comments:
Date of review: ___/___/______ I have achieved these goals (circle a number): 1 (Not at all) 2 3 (A little) 4 5 (Very well)	Comments:

Worksheet: Week 2

**Write your goal here:** ______________________ **Start date:** ___/___/______	Comments:
Date of review: ___/___/______ I have achieved these goals (circle a number): 1 (Not at all) 2 3 (A little) 4 5 (Very well)	Comments:

Worksheet: Week 3

**Write your goal here:** ______________________ **Start date:** ___/___/______	Comments:
Date of review: ___/___/______ I have achieved these goals (circle a number): 1 (Not at all) 2 3 (A little) 4 5 (Very well)	Comments:

REMINDER: For every week, you work towards the new goal for that week and all previous goals. For example, in week 2 you record achievement of goal 1 and goal 2, and in week 5 you record goals 1-5.

INTEREST_Booklet_1.0_170817 IRAS Project ID 228033

Environmental Modification Creation Process

Question	Example Answer(s)	Example Modification(s)
Where do I sit the most?	1. On the sofa in front of the TV<br>2. In the garden<br>3. In the kitchen<br>4. In the living room	1a. Keep the TV remote at the TV itself rather than next to the sofa so that I must stand up to get it<br>1b. Use an electrical device that switches the TV off between specific times<br>2. Remove the garden chairs in the afternoon so that time spent in the garden should be standing or gardening<br>3. Remove the chairs from the kitchen in the afternoon<br>4. Put up prompts on the walls of the living room or on the kitchen table (or any other room) to remind me to stand up when I notice them
How long do I sit?	I sit for multiple hours at a time	Use a pedometer or other appliance that encourages me to get a certain amount of steps per day— we can supply you with this (some phones can also do it)
Why don't I walk enough?	I don't feel motivated to get up and go outside	Start the day by wearing my outdoor clothes, which will make me more likely to leave the house

Hopefully these examples have given you a good idea of how to formulate your goals. In the following pages, you'll be given space to make six goals of your own and three environmental modifications in the same format as this table. Each should be specific to your own lifestyle and situation, but feel free to take some inspiration from the examples given if they relate to your own life. As in this example, it is recommended to use the first three goals to relate to the top three contexts in which you sit for prolonged periods. For this reason, those questions are already filled in for you. There is enough room to come up with a couple of options for each goal, but remember you need six goals and three environmental modifications in total to cover six weeks. Please ask the researcher if you have any questions or need inspiration.

Additional advice

This following section of this booklet will help you finalise your goals to adhere to a certain number of principles, known as the SMART principles. Secondly, it will allow you to record your goals and environmental modifications so that you can also write down how well you achieve them throughout the course of this study.

You probably already have some goals in mind based on the previous pages of this booklet. However, your goals may still be a little unclear, for example they may lack specificity to certain contexts, or you may not know exactly when, where, or how you're supposed to be achieving them. A system known as the SMART system will help to make sure that your goals are relevant to several criteria. To ensure your goals are as precise as possible, you must make sure how it is written covers all of the components of SMART. Please see below for a description of each element of the SMART system.

Specific	Your goals ought not be too vague. You must know exactly in which context you are supposed to be achieving the goal. It should not be a general statement.
Measurable	This means that the goal must be formulated so that you can know when you have successfully achieved the goal.
Achievable	The goal should not be too difficult, and the context which it relates to should occur often enough in your daily life, so that you know that achieving this goal helps you sit less overall.
Relevant	Your goals need to be related to one specific context you encounter in your day to day life. For example, sitting down when reading a book.
Timely	Your goals will already be set to be relevant on a specific week. However, you should know at which times of day you should be achieving the goal as well.

To use the SMART system properly, you must make sure that each of your goals adhere to each of the individual parts of SMART. This will mean you know exactly what to do, how to do it, where to do it, and when. Therefore, you will definitely be reducing the amount of time you spend sitting in no time! Remember: your goals can relate to reducing the total amount of time sitting, or to increasing how frequently you go from sitting to standing. All of this will help you get into better shape for surgery. In the following pages, you'll find space to write your goals and the modifications you make to your home environment and how well you achieve them. Please remember to complete it at the end of each week.

Goal Creation Worksheet

Question	Answer	Goal
What activity do I do most when sitting?		1.
What is the second most common activity I perform whilst sitting?		2.
What is the third most common activity I am doing when sitting?		3.
		4.
		5.
		6.

Environmental Modification Worksheet

Question	Answer	Modification
		1.
		2.
		3.

Once you've formulated your goals, please transfer them onto the following pages for each relevant week. Put the goals which are likely to be most impactful in the earlier weeks, and the goals which are more specific in the later weeks.
