# Supplementary material for: A novel behavioural INTErvention to REduce Sitting Time in older adults undergoing orthopaedic surgery (INTEREST): protocol for a randomised controlled feasibility study
Source: Pilot Feasibility Stud. 2019 Apr 6;5:54. doi: 10.1186/s40814-019-0437-2 (PMC6451782; doi:10.1186/s40814-019-0437-2)
Supplement: Supplementary file 2 — Theoretical development of INTEREST. (DOCX 49 kb) [file 40814_2019_437_MOESM2_ESM.docx]

# Theoretical Development of INTEREST

Changing human behaviour is always a difficult endeavour and changing the well-engrained habits of older adults dealing with the burden of morbidity, as is the case here, is especially challenging. When developing complex interventions, it is considered best-practice to establish a clear logic model and explicit theoretical underpinnings to allow for explanation of the mechanisms of action [1]. However, a recent review of behaviour change strategies to reduce sedentary behaviour in adults found that 58% of included interventions (15 out of 26) did not mention a theoretical framework [2]. Of those that did, seven mentioned using a Transtheoretical Model, four used Social Cognitive Theory, three used the Theory of Planned Behaviour, and one used Empowerment theory [2]. Despite this lack of clearly defined theory, interventions were found to generally assume that sedentary behaviour is largely determined by the external environment, or problems with self-regulation. However, as Gardner et al. (2015) point out, although interventions included in their review, none gave any attention to the construct of motivation when attempting to change behaviour [2].

A recent systematic review of interventions in older adults extracted the theoretical frameworks used in the included interventions [3]. Of the six included studies, one did not include a theoretical framework, and each of the five others used a different theory. These included empowerment theory, social cognitive and behavioural choice theories, the health action process approach, a habit dual-process framework, and a habit formation model. However, the most promising trial reviewed used a multi-componential design based upon Self-Determination Theory (SDT). This theory emphasises motivation as being key to behaviour – a missing consideration in many sedentary behaviour interventions developed to date (Gardner et al., 2015).

## Application of Self-Determination Theory

SDT has been found to have great success when used in other sedentary behaviour and exercise interventions. To maximise behaviour change, SDT states that intrinsic motivations (including self-generated motivation or internalisation (integration) of externally-generated instructions or ideas) are more powerful than extrinsic (externally generated, but not internalised) motivations [4, 5]. Fundamental to the generation of intrinsic motivation are three psychological needs proposed by Ryan & Deci (2000) in their Basic Psychological Needs theory. These three needs; competence, autonomy, and psychological relatedness, serve as useful targets for enhancement when designing interventions. Competence refers to a desire to feel control over the outcomes of one’s own actions; relatedness is the desire to connect to and be approved of or accepted by others, and autonomy is the desire to be the agent of one’s self (Ryan & Deci, 2000). Enhancing these needs leads to greater internalisation of goals into “integrated regulatory processes”, greater personal growth and development, and the resulting accomplishment of one’s own goals.

Logic model and overall component combination

#### Explaining the Logic Model

Figure 2 in the main manuscript depicts the overall rationale for the implementation of SDT in INTEREST. As discussed, the components themselves are designed to target specific basic psychological needs. In figure 2, the primary target for each component is shown, although some, such as individualised goal-setting, would likely enhance both competence and autonomy. In figure 2, in line with Organismic Integration Theory, satisfaction of the Basic Psychological Needs moves individuals along a spectrum towards integrated regulation of the new behaviours introduced as part of the study. By actively achieving their goals, participants will be expected to gain feelings of achievement, leading to greater persistence of behaviour change and enhanced well-being.

# Rationale for intervention components

All the intervention components in INTEREST, outlined in the prior section, were chosen for their integration within Self-Determination Theory. All the chosen components have a strong literature base which will be further outlined in this section.

## Supporting the basic psychological needs (person-centred delivery style)

As the Basic Psychological Needs are integral constructs within Self-Determination Theory, the intervention is based upon the concept that supporting autonomy, competence, and relatedness within participants will lead to maximised motivation. The primary is on supporting participant autonomy, as a review of 32 interventions for problem drinkers found that the most important determinants of change were giving nonevaluative feedback, encouragement of participant responsibility, and empathetic delivery, all of which are indicative of a person-centred delivery style [6]. Healthcare practitioners that create controlling contexts have also been found to lead patients on a path towards introjection rather than integrated regulation of new behaviours, meaning that motivational processes will not be maximised [7].

Phone calls are integrated as an important component of the intervention to engage with participants in a person-centred manner, to enhance motivation and feelings of relatedness. This is supported by findings from Deci (1971), who found that giving people unexpected positive feedback not only acts as a reward, but also increases feelings of competence, thus increasing intrinsic motivation and reinforcing the behaviour. Therefore, during the phone calls, positive encouragement will be given.

## Motivational interviewing (MI)

Motivational interviewing and SDT share many fundamental assumptions [9]. Motivational interviewing is primarily a client-centred counselling technique aimed at reducing a patient’s ambivalence about making a change, primarily by supporting their agency and helping them change of their own accord, by mobilising a patient’s own internal motivations [10]. Both SDT and MI emphasise the role of personal autonomy as a driving force of human behaviour [9]. However, one of the authors of SDT has stated that motivational interviewing’s similarities with SDT are primarily dependent upon the manner with which it is delivered [11]. Just like with spousal or familial support, MI only integrates well with SDT when the practitioner can deliver it in an autonomy-supportive manner.

According to a review of 15 studies investigating the efficacy of MI in older adult populations with acute and chronic illnesses, whether to improve physical activity participation or quit smoking or alcohol, or a combination of lifestyle behaviours, MI was found to be effective at increasing PA, reducing smoking and alcohol behaviours, and improving health markers [12]. However, results were conflicting about whether behaviour change persists long-term [12].

MI has been successfully employed once before in a RCT of n=56 community-dwelling older adults of 65 years and above (95.7% women), living with chronic pain in Hong Kong, with the intention of reducing their pain and increasing their participation in physical activity [13]. MI was delivered in a group setting for 30 minutes weekly over the course of 8 weeks. The pain intensity at baseline was not significantly different between groups, but after intervention and MI delivery, 8 weeks later, pain was significantly reduced in the intervention group as compared to the control [13]. This study demonstrates that MI has been used appropriately and has been found to be effective in community-dwelling older adults with chronic lower body pain. MI has also been used in an intervention to reduce sedentary time in n=23 frail older adults in Scotland, however they were not able to reduce sedentary time in this group [14].

Overall, MI was incorporated into the design of INTEREST due to its excellent history for integrating well with SDT, and due to its demonstrated record of efficacy.

## Individual feedback on walking, standing, and sitting behaviour

According to a review which taxonomized different types of feedback in health behaviour interventions, there are three main types of feedback [15]. The first is generic feedback, which is relevant to an entire population. Next, there is targeted feedback, based on demographic characteristics. Finally, there is personalised feedback, which provides specific information for an individual based on either normative (relating to the ‘normal’ for others) or ipsative (comparing someone to their prior performance) comparisons. In INTEREST, the feedback given will be personalised and ipsative, as it could be considered unfair to compare osteoarthritic individuals with healthy older adults, or even other osteoarthritic older adults, due to the heterogeneity of the condition. Participants are likely to have significant ranges in physical function and could have had different severities of osteoarthritis or other co-morbidities, which means that normative comparisons could be unattainable and discouraging for some.

Individual feedback has been shown to be effective in other behaviour change interventions, such as one to increase hand hygiene behaviours in n=24 nurses [16]. Behaviour was monitored with direct observation, and participants were given a score based on their individual frequency of handwashing in comparison to the required amount of handwashing. Hand washing quality was also assessed, by making bacterial cultures from each person’s right hand after handwashing and showing these to participants. In combination with education about handwashing, feedback on handwashing behaviour was able to increase handwashing frequency from 46.8% to 71.4% [16]. Given the efficacy of this approach and its utility to goal-setting, personalised feedback is an integral component of the INTEREST design.

## Individualised, incremental goal setting

Goal-setting has been used in hundreds of behaviour change interventions, and a recent meta-analysis of 141 papers has found it to be highly effective, particularly so when the goal was challenging to achieve, set in a group, or publicly [17]. Originating in Goal Setting Theory, goal setting has been used to change behaviour in almost all domains [17, 18]. For example, a combination of personalised feedback and individualised goal setting has been used to increase weekly running distance in healthy adults [19] and to aid obese older adults in weight loss and to prevent weight regain [20]. Goal Setting Theory maintains that goals should be conscious and specific, rather than vague, such as “do x when y occurs”, rather than “be better at x” [17]. Four modifiers are postulated that enhance behaviour change as a result of goal setting, namely (1) intention to adhere to the goal, (2) low degree of complexity of the goal (not the same as difficulty), (3) feedback about progress towards the goal, and (4) adequate resources available/few constraints [17, 21]. Goal Setting Theory originally did not consider older adults as a specific use case, however, since then, the authors have elaborated that older adults have a number of specificities that synergise quite well with goal setting [17].

However, the approach in this study adds two other aspects, inspired partly by SDT; namely, the individualised and incremental aspects of goal setting. These operate upon two main assumptions: firstly, that by individualising the goals, the goals are more specific to an individual’s lifestyle and thus more achievable for them, again enhancing their autonomy; and, secondly, that by incrementally introducing them over the course of weeks it would be less difficult for people to adjust to their goals as it would be less cognitively demanding and impactful on people’s lifestyles. In addition to being personalised, the goals will be created using SMART principles, which were originally developed for application in a business context and have since expanded to healthcare contexts as a way to make behaviour change more personalised and manageable in a real-world context [22, 23]. SMART stands for Specific, Measurable, Achievable, Relevant, and Time-specific [23]. By ensuring they are SMART, the goals will fulfil the suggestions of the systematic review above, namely that the goals are formulated with the intention of adherence and ensuring availability of enough resources to achieve them. Finally, in combination with the self-monitoring that is to be discussed later in this chapter, the participants will be able to self-feedback on their own goal achievement, especially in combination with the weekly goal adherence monitoring in the booklet.

## Education about sedentary behaviour

As the field of sedentary behaviour research is relatively new, it is very possible that older adults are not aware of the potential health ramifications of sitting. A recent study found that only 14 percent of older adults 55 aged years or over in the UK had accurate knowledge about physical activity guidelines [24]. Given these low figures regarding the better-established guidelines around physical activity, knowledge about the health effects of sedentariness is likely to be even lower. Although education by itself is not sufficient to drive behaviour change, informing participants about the health impact of sitting, it may be feasible for this to serve as additional motivation for them to sit less [25].

## Environmental modification

The built environment is acknowledged in a large number of behavioural theories as a key determinant of behaviour, and has been found to be a key driver of sedentariness as well [26, 27]. Therefore, environmental modification as a tool for behaviour change has been identified by reviews to be a highly promising in sedentary behaviour interventions [2]. Environmental modification has also been used in other interventions to good effect, such as in interventions to drive eating behaviour change to elicit weight loss [28, 29]. A recent RCT found that a behavioural weight loss program was less effective than a behavioural weight loss program combined with environmental modification, in which modifications included providing participants with exercise equipment in the home, giving them scales and full-body mirrors, health focused magazines, and incorporating partner support [29]. Interestingly, environmental modification has also been used to reduce TV viewing behaviour, a very common form of sedentary behaviour, in a pilot study, by turning off power to TVs after an allotted time period expires [30]. We included this BCT in INTEREST, as by modifying home environments, such as by putting up posters to remind them of their goals and aspirations, participants will be able to create a more autonomy-supporting and competence-supporting environment.

## Self-Monitoring and Self-Regulation

Self-monitoring is a technique that involves use of self-regulatory behavioural processes and is often considered to be an energy-consuming process that is fatiguing for the person doing it [31]. However, it has been found that when self-regulation is being practised based upon a foundation of autonomous choice, rather than controlled regulation (i.e. it is self-regulation for a behaviour the person *wants* to do), then it is actually not an energy-draining task for the individual [32].

This self-monitoring will be likely to increase engagement and feelings of self-control when working towards achieving their behavioural targets and enhance self-regulatory abilities, and a sense of achievement will aid in self-efficacy.

## Support of spouse, family, friends, and the practitioner

In healthcare interventions, it is integral for the concept of relatedness for the client/patient to feel that they are respected, understood, and cared for, by the practitioner and by their family and friends [33]. Without these factors in place, it is less likely that the patient will be likely to accept and want to work toward recommendations of a healthcare practitioner. Within the framework of SDT, social factors can have both positive and negative effects in terms of the satisfaction of the basic psychological needs [34]. Social support can be either autonomy-supportive, or controlling, in nature, however, the former has been found to be supportive of the basic psychological needs of competence and relatedness [34]. Social support has been found to be generally autonomy-supportive, and therefore beneficial to be included in interventions [34, 35].

## Problem solving, progress monitoring, and setback management

These aspects of the intervention will be delivered during phone calls by the researcher. These BCTs have not been explored for their integration into SDT, however, the usage of these techniques will be in an autonomy-supportive, person-centred style and will aim to also place emphasis on the competence of the participant.

# References

1. Craig P, Dieppe P, Macintyre S, Michie S, Nazareth I, Petticrew M. Developing and evaluating complex interventions: the new Medical Research Council guidance. BMJ. 2008;337:a1655.

2. Gardner B, Smith L, Lorencatto F, Hamer M, Biddle SJ. How to reduce sitting time? A review of behaviour change strategies used in sedentary behaviour reduction interventions among adults. Health Psychol Rev. 2015;7199 October:1–24. doi:10.1080/17437199.2015.1082146.

3. Aunger JA, Doody P, Greig CA. Interventions targeting sedentary behavior in non-working older adults: a systematic review. Maturitas. 2018. doi:https://doi.org/10.1016/j.maturitas.2018.08.002.

4. Ryan R, Deci E. Self-determination theory and the facilitation of intrinsic motivation. Am Psychol. 2000;55:68–78. doi:10.1037/0003-066X.55.1.68.

5. Silva MN, Vieira PN, Coutinho SR, Minderico CS, Matos MG, Sardinha LB, et al. Using self-determination theory to promote physical activity and weight control: a randomized controlled trial in women. J Behav Med. 2010;33:110–22. doi:10.1007/s10865-009-9239-y.

6. Bien TH, Miller WR, Tonigan JS. Brief interventions for alcohol problems: a review. Addiction. 1993;88:315–36.

7. Williams GC, Deci EL, Ryan RM. Building health-care partnerships by supporting autonomy: Promoting maintained behavior change and positive health outcomes. Partnerships Healthc Transform relational Process. 1998;:67–87.

8. Deci EL. Effects of externally mediated rewards on intrinsic motivation. J Pers Soc Psychol. 1971;18:105.

9. Markland D, Ryan R, Tobin V, Rollnick S. Motivational Interviewing and Self-Determination Theory. J Soc Clin Psychol. 2005;24:811–31.

10. Letourneau K. A patient-centered approach to addressing physical activity in older adults. J Gerontol Nurs. 2014;40:26–32.

11. Deci EL, Ryan RM. Self-determination theory in health care and its relations to motivational interviewing : a few comments. Int J Behav Nutr Phys Act. 2012;9:1–6.

12. Cummings SM, Cooper RL, Cassie KM. Motivational Interviewing to Affect Behavioral Change in Older Adults. 2009;:195–204.

13. Tse MMY, Vong SKS, Tang SK. Motivational interviewing and exercise programme for community-dwelling older persons with chronic pain : a randomised controlled study. 2013;:1843–56.

14. Harvey JA, Chastin SFM, Skelton DA. Breaking sedentary behaviour has the potential to increase / maintain function in frail older adults. 2018;3:26–34.

15. Diclemente CC, Marinilli AS, Singh M, Bellino LE. The Role of Feedback in the Process of Health. Am J Heal Behav. 2001;:217–27.

16. Chun H-K, Kim K-M, Park H-R, Hee K, Korea S. Effects of hand hygiene education and individual feedback on hand hygiene behaviour, MRSA acquisition rate and MRSA colonization pressure among intensive care unit nurses. Int J Nurs Pract. 2014;21:709–15. doi:10.1111/ijn.12288.

17. Epton T, Currie S, Armitage C. Unique effects of goal setting on behavior change: Systematic review and meta-analysis. J Consult Clin Psychol. 2017;Forthcomin:1182–98.

18. Locke EA, Latham GP. A theory of goal setting & task performance. Prentice-Hall, Inc; 1990.

19. Wack SR, Crosland KA, Miltenberger RG. Using goal setting and feedback to increase weekly running distance. 2014;1:181–5.

20. Nicklas BJ, Gaukstern JE, Beavers KM, Newman JC, Leng X, Rejeski WJ. Self-monitoring of spontaneous physical activity and sedentary behavior to prevent weight regain in older adults. Obes. 2014;22:1406–12. doi:10.1002/oby.20732.

21. Locke EA, Latham GP. New Directions in Goal-Setting Theory. Curr Dir Psychol Sci. 2006;15:265–8. doi:10.1111/j.1467-8721.2006.00449.x.

22. Shaw RL, Pattison HM, Holland C, Cooke R. Be SMART : examining the experience of implementing the NHS Health Check in UK primary care. 2015;:1–8.

23. Doran GT. There’s a S.M.A.R.T. way to write management’s goals and objectives. 1981.

24. Knox ECL, Esliger DW, Biddle SJH, Sherar LB. Lack of knowledge of physical activity guidelines: can physical activity promotion campaigns do better?: Table 1. BMJ Open. 2013;3:e003633. doi:10.1136/bmjopen-2013-003633.

25. Michie S, Van Stralen MM, West R. The behaviour change wheel: a new method for characterising and designing behaviour change interventions. Implement Sci. 2011;6:42.

26. Owen N, Salmon J, Koohsari MJ, Turrell G, Giles-corti B. Sedentary behaviour and health : mapping environmental and social contexts to underpin chronic disease prevention. 2014;:174–7.

27. Sallis JF, Owen N, Fisher E. Ecological models of health behavior. Heal Behav Theory, Res Pract. 2015;5:43–64.

28. Carels RA, Young KM, Koball A, Gumble A, Darby LA, Wagner Oehlhof M, et al. Transforming your life: An environmental modification approach to weight loss. J Health Psychol. 2011;16:430–8.

29. Gorin AA, Raynor HA, Fava J, Maguire K, Robichaud E, Trautvetter J, et al. Randomized controlled trial of a comprehensive home environment-focused weight-loss program for adults. Heal Psychol. 2013;32:128–37.

30. Gorin A, Raynor H, Chula-Maguire K, Wing R. Decreasing household television time: a pilot study of a combined behavioral and environmental intervention. Behav Interv. 2006;21:273–80.

31. Bandura A. The primacy of self regulation in health promotion. ApplPscyhIntRev. 2005;54:245–54.

32. Moller AC, Deci EL, Ryan RM. Choice and ego-depletion: The moderating role of autonomy. Personal Soc Psychol Bull. 2006;32:1024–36.

33. Ryan RM, Patrick H, Deci EL, Williams GC. Facilitating health behaviour change and its maintenance: Interventions based on self-determination theory. Eur Heal Psychol. 2008;10:2–5.

34. Deci EL, Ryan RM. Motivation, personality, and development within embedded social contexts: An overview of self-determination theory. Oxford Handb Hum Motiv. 2012;:85–107.

35. Deci EL, La Guardia JG, Moller AC, Scheiner MJ, Ryan RM. On the benefits of giving as well as receiving autonomy support: Mutuality in close friendships. Personal Soc Psychol Bull. 2006;32:313–27.
