# Supplementary material for: A novel behavioural INTErvention to REduce Sitting Time in older adults undergoing orthopaedic surgery (INTEREST): protocol for a randomised controlled feasibility study
Source: Pilot Feasibility Stud. 2019 Apr 6;5:54. doi: 10.1186/s40814-019-0437-2 (PMC6451782; doi:10.1186/s40814-019-0437-2)
Supplement: Supplementary file 3 — INTEREST Fidelity Toolkit. Document detailing the fidelity assessment processes in the INTEREST study. (DOCX 48 kb) [file 40814_2019_437_MOESM3_ESM.docx]

INTEREST Fidelity Toolkit

The INTEREST fidelity measure for components delivered by a facilitator is a six-point scale measuring adult skill acquisition to reflect the competence level of intervention component delivery (Dreyfus, 2004). This scale ranges from 0, indicating an absence of skill, up to 5, which would be considered an ‘expert’ level of delivery.

##### Table 1. The generic (non-applied) 6-point score of adult skill acquisition (Dreyfus, 2004).

| **Competence Level** | **Scoring** | **Description** |
| --- | --- | --- |
| Absence | 0 | Does not resemble the skill attempting to be used. |
| Novice | 1 | Minimal use of the skill and /or inappropriate performance of the skill. |
| Advanced Beginner | 2 | Evidence of competence, facilitator begins to understand the context involved, but numerous problems and inconsistencies. Detached involvement. |
| Competent | 3 | Competent, aware of and able to cope with different contexts and situations, but with minor problems. More engaged involvement. |
| Proficient | 4 | Minimal problems or inconsistencies. Able to adapt appropriately to a variety of contexts and situations. Involved understanding. |
| Expert | 5 | Highly appropriate and proficient with no problems. Able to make more subtle refined discriminations between contexts and able to adjust accordingly. Highly involved understanding. |

At any time, the delivery may encounter difficulties, for example due to resistance from the participant, etc. However, in these cases the assessor should still focus on rating the delivery of the facilitator, whatever the response of the client may be.

## Performance rating process

Separate documents are available for the rating of individual sessions in the intervention (visit 2/3, phone calls, and action plan rating). Using these forms, the initial assessment should consider whether the key features of the skill are present (e.g. for motivational interviewing, does the facilitator use OARS, etc.). Secondly, assess whether these skills are delivered in the appropriate contexts rather than merely being present. If a facilitator both utilises all the required skills, and in the appropriate contexts (i.e. doesn’t miss many opportunities nor misuses opportunities), then they would be rated among the top competencies. This document covers motivational interviewing, problem solving, progress monitoring, setback managing, and action planning skills.

## Performance standards

The intervention will be considered as having been delivered to a good standard if the mean competence of delivery is at level 3 or greater across all items, and if action plans score a 3 or above.

# Item 1: Motivational interviewing

### Key features

The Motivational Interview (MI) should be delivered in a client-centred manner, encouraging the patient to be the main driver of behaviour change in an *autonomous* fashion. The instructor should not be dictatorial, judgemental, or disrespectful. Rather, they should *support the patient* and provide them with resources and *encouragement* with which they can realise their own capacity for change. The interaction should demonstrate *a genuine sense of empathy and warmth* from the facilitator and should be *individually tailored* to the patient’s specific needs and should *adapt* in response to new information. The patient should be talking for *more than half of the time*. The facilitator can share their own experiences and provide *information/expertise*, but only in a manner that asks *permission*, and is *open-ended* and not dictatorial. MI techniques will also be used in the phone calls where appropriate, and the below skill table (Table 2) may also be used to aid in skill rating after the phone calls where required.

### Intervention techniques

1. In the initial phases of the MI (engaging, focusing), OARS (open questions, affirmation, reflective listening, summaries) should be used throughout to guide the discussion.
2. Reflective listening at minimum consists of simple reflections that repeat or rephrase elements of what the patient said, but a highly skilled facilitator will use complex reflections that exaggerate/amplify the reflections, reframe, emphasise personal autonomy, or reinforce key theoretical components of the intervention /logic model (e.g. highlighting participant experiences of relatedness or their growing sense of competence).
3. Change talk should be elicited from the patient, and then the deliverer should switch to EARS (elaborating, affirming, reflecting, and summarising).
4. Summaries should be used throughout that reinforce/affirm/praise patient effort.
5. The responses of the facilitator should exhibit specific tailoring to the responses of the patient and there should be evidence of a collaborative relationship. There should be no evidence of arguing, disagreements, judgment, blame, or persuasion.

##### Table 2: MI checklist

Place an X in the box for the highest competency level for which the facilitator fulfils all criteria.

| **Score** | **Proficiency** | **Description of characteristics** |
| --- | --- | --- |
|  | 0 – Absence of skill | An overly directing, practitioner-led, or dictatorial style of interaction without any evidence of change talk. |
|  | 1 – Novice | Little patient involvement. Minimal evidence of use of MI techniques. The facilitator talks for most of the session. |
|  | 2 – Advanced beginner | Some evidence of MI technique usage. Facilitator dominates the discussion. Numerous problems or inconsistencies. Detached involvement. |
|  | 3 – Competent | Appropriate use of basic MI techniques (OARS) and summaries. Change talk becomes evident. Evidence of a collaborative relationship. Difficulties in content or method of delivery. |
|  | 4 – Proficient | Exclusive use of client-centred delivery style. Participant and facilitator have a collaborative relationship. Little difficulty and few missed opportunities to use MI techniques. Some use of advanced MI skills, such as complex reflections, and summaries are delivered where appropriate, in a manner that furthers the discussion. |
|  | 5 – Expert | Highly proficient use of a wide range of advanced MI techniques, e.g. complex, strategic reflections, summaries, etc. No opportunities missed or evidence of problems. Smoothly transitions with new information and uses it in the MI techniques. |
|  | N/A | Not relevant to this activity. |

# Item 2: Phone call delivery

### Key features

The phone calls in INTEREST serve the following purposes:

1. To serve as a motivational tool if the patient shows signs of reverting to a less motivated or more ambivalent state. This is on a case-by-case basis (where required).
2. To query the patient about goal adherence and offer opportunities for problem-solving collaboratively with the patient if any goals/environmental modifications are deemed as unachievable. This should result in changes to the goals to make them achievable (where required).
3. To monitor progress.
4. To manage setbacks (where required).

Given that many techniques are used, assessment of fidelity of this area will require a number of techniques to be applied, including MI techniques (item 1) and problem-solving skills (item 2a), progress monitoring skills (item 2b), and setback management skills (item 2c).

### Intervention techniques

The facilitator should appropriately identify in which phone calls MI techniques are required, and pursuant to that, use problem solving skills as appropriate to work with the patient on a solution any problems that they have identified, monitor progress, and help manage setbacks (where needed).

Due to the private nature of these calls, the practitioner will have to engage in a self-rating exercise soon after the call is completed (Table 3).

## Item 2a: Problem-solving

### Intervention techniques

Reframing should be used in cases where there’s a setback to focus on the opportunity to use it as a learning experience and help support the patient. Goals should be revised and reformulated where necessary as part of problem solving and progress monitoring. Techniques such as identifying barriers; breaking problems down (into easier chunks) and exchanging information in an ask-tell-discuss manner (e.g. to address misconceptions or to stimulate ideas for overcoming barriers) will be utilised.

##### Table 3: Problem solving checklist

| **Score** | **Proficiency** | **Example** |
| --- | --- | --- |
|  | 0 – Absence of skill | Absence of discussion to suggest appropriate problem-solving strategies relating to the action plan. |
|  | 1 – Novice | Minimal discussion to suggest appropriate problem-solving strategies relating to the action plans and/or inappropriate delivery. Amendments to action plans are not made or made poorly despite being required. |
|  | 2 – Advanced beginner | Only a small part of the discussion is delivered to a competent level. Some discussion to suggest appropriate problem-solving strategies relating to the action plans, however, these may not be carried out to sufficient depth or detail. Adapting appropriately to context involved, but numerous problems and inconsistencies present. Detached involvement. Amendments to action plans are made poorly. |
|  | 3 – Competent | Competent and numerous discussions to suggest appropriate problem-solving strategies relating to prior action plan, however some difficulties are evident (e.g. opportunities to discuss missed, not all areas of problem covered). Able to cope with different context and situations. Minor problems or inconsistencies present. More engaged involvement. Action plans are amended satisfactorily where required, but problems evident, e.g. goals are no longer SMART. |
|  | 4 – Proficient | Numerous discussions to suggest appropriate problem-solving strategies with respect to the action plan, able to discriminate between a variety of contexts and situations with some minor problems or inconsistencies evident. Action plans are amended effectively where required. |
|  | 5 – Expert | Highly appropriate suggestion(s) of appropriate problem-solving strategies with respect to problems with the prior action plan. Able to make more subtle refined discriminations between contexts and able to adjust accordingly. Minimal or no discernible problems. Action plans amended without any problems. |
|  | N/A | Not relevant to this activity. |

##### Goal amendment table

| **Old Goal or EnviroMod number** | **New Goal or EnviroMod** | **Reason for Amendment** |
| --- | --- | --- |
|  |  |  |
|  |  |  |
|  |  |  |
|  |  |  |
|  |  |  |
|  |  |  |
|  |  |  |
|  |  |  |
|  |  |  |

## Item 2b: Monitoring progress

### Intervention techniques

The facilitator should ask about progress against the action plans made, actively explore areas in which the patient has experienced benefits as a result of their behaviour change and seek to reinforce these benefits in order to help maintain patient motivation and achievement. The facilitator should also encourage ongoing self-monitoring of the targeted behaviours.

##### Table 4: Monitoring progress checklist

| **Score** | **Proficiency** | **Example** |
| --- | --- | --- |
|  | 0 – Absence of skill | Absence of discussion to monitor participant progress on the action plan. |
|  | 1 – Novice | Minimal or inappropriately delivered discussion to monitor participant achievement on the action plan. |
|  | 2 – Advanced beginner | Some evidence of competence. Some discussion to monitor participant progress towards achievement of the action plan, however, not in sufficient detail or depth. Detached involvement. |
|  | 3 – Competent | Competent and numerous discussions to monitor participant progress towards the action plan, however difficulties are evident (e.g. opportunities to discuss missed, not covering all aspects of the problem). Competent, aware of and able to cope with different contexts and situations. More engaged involvement. |
|  | 4 – Proficient | Involved discussion to monitor participant on the action plan, some minor problems or inconsistencies evident. |
|  | 5 – Expert | Highly appropriate and sufficient discussions monitoring participant progress towards the action plan. Minimal problems. |
|  | N/A | Not relevant to this activity. |

## Item 2c: Managing setbacks

### Intervention techniques

Setbacks should be managed using reframing techniques, to change perspectives to allow for viewing of failures as opportunities for change. Participants should be informed about coping plans as a strategy for managing the setbacks, to aid in the sustainability of change.

##### Table 5: Managing setbacks checklist

| **Score** | **Proficiency** | **Example** |
| --- | --- | --- |
|  | 0 – Absence of skill | Absence of discussion to review participant setbacks relating to action plans and/or highly inappropriate performance. |
|  | 1 – Novice | Minimal (or poorly delivered) discussion to review action plans and/or inappropriate performance. |
|  | 2 – Advanced beginner | Some evidence of competence. Some discussion to review participant setbacks relating to physical activity behaviours however, these may not be carried out to sufficient depth or detail. Adapting appropriately to context involved, but numerous problems and inconsistencies. Detached involvement. |
|  | 3 – Competent | Competent and numerous discussions to review participant setbacks in achievement of action plans. However, some difficulties are evident, such as minor inconsistencies. More engaged involvement. |
|  | 4 – Proficient | Numerous discussions to review participant setbacks when achieving action plans, able to discriminate between a variety of contexts and situations with some minor problems or inconsistencies evident. Some discussion of coping plans. |
|  | 5 – Expert | Highly appropriate and sufficient review of participant setbacks relating to achievement of action plans. Able to make more subtle refined discriminations between contexts and able to adjust accordingly. Minor or no discernible problems. Clear discussion of coping plans. |
|  | N/A | Not relevant to this activity. |

# Item 3: Supporting the Basic Psychological Needs

### Key features

The INTEREST study was designed using the theoretical framework of Self-Determination Theory (SDT). Within SDT is basic psychological needs theory, which states that we all have three key needs: autonomy, competence, and relatedness. Individuals will maximally achieve behaviour change when these basic needs are most fulfilled. Thus, within the study, all activities should be supportive of the patient’s basic psychological needs.

### Intervention techniques

To enhance the basic psychological needs throughout the intervention, opportunities should be taken to enhance participant autonomy by emphasising patient choice (i.e. by emphasising that each of the goals are their choice), highlighting their strengths and agency, and supporting them to overcome their own barriers and to achieve their goals. Competence should be supported as well, by providing them with the tools they need to feel a sense of achievement, by recognising efforts towards achieving goals, by supporting change talk, and praising participant choices and achievements. Likewise, relatedness can be aided by fostering an environment in which social interaction can occur, by encouraging spousal/familial involvement where possible, and by supporting or suggesting activities that occur in a social context. This includes addressing any negative social influences on achievement of the action plans.

###### Table 6: Supporting Basic Psychological Needs checklist

| **Score** | **Quality level** | **Description of characteristics** |
| --- | --- | --- |
|  | 0 – Absence of skill | None of the basic psychological needs were supported. |
|  | 1 – Novice | Slight support for one of the basic needs. |
|  | 2 – Advanced beginner | The patient was supported in most of the basic needs to some degree, however many opportunities were missed for reinforcement, encouragement, etc. |
|  | 3 – Competent | All of the basic psychological needs were supported, but some opportunities to support the patient were missed. |
|  | 4 – Proficient | Collaborative relationship between deliverer and patient was clear, and autonomy was highly supported. Few opportunities missed to emphasise autonomy, enhance relatedness, or to foster competence. |
|  | 5 – Expert | All needs were supported, no opportunities missed. |

# Item 4: Formulation of an appropriate action plan (action planning)

As the action plans are not purely reliant upon the skill of the deliverer, but also reliant upon the participant having a good understanding of their own context and behavioural patterns, the goal-plan will be assessed in two stages. Firstly, quality of support for the action-planning process will be self-rated by the facilitator using a measure based on the six-point scale of adult skill acquisition (Dreyfus, 2004) (Table 7). Secondly, plan content and quality will be assessed retrospectively at the end of the study. This will be conducted with a custom-designed measure of plan content and quality according to the requirements of INTEREST (Table 8), and not the six-point scale of adult skill acquisition.

### Key features

The facilitator should work with the participant to formulate an effective set of 6 goals and 3 environmental modifications with which the patient can reduce their sedentary behaviour and increase their movement. All the goals and environmental modifications should clearly target an aspect of sedentary behaviour or a behaviour with which sedentariness is commonly displaced: namely, they should be related to one of the following:

- Reduction in total sitting time.
- Reduction in the average length of sedentary bouts/greater frequency of breaks in sitting.
- Increased standing behaviour.
- Increased walking.
- Increased quantity of sit-to-stand transitions.
- Increase some other kind of physical activity.

The action plan should also be appropriate to the level of physical function of the participant. The action plan can be cross-referenced to the physical function or Short Physical Performance Battery (SPPB) score to ensure that the plan is suitably individualised. Each of the goals should adhere to SMART principles (i.e. be specific, measurable, achievable, realistic, and timely).

To rate the action plan, the assessor should first rate each of the goals made for a single participant according to the SMART principles using Table 8 below. Using Table 9 while looking at the whole of the action plan, plus the scoring in Table 8, the assessor will then be able to rate the overall level of quality of the action plan as a whole. Action plans will be considered as delivered to a good level if the average score across the intervention is a 4 or above. Some SMART items, such as whether goals are achievable and realistic may have to be assumed or related also to the physical function level of the participant.

### Intervention techniques

The facilitator should ensure that the action plan is made as a collaborative process that is participant-focused and is supportive of their autonomy. The patient should be heavily involved in the formulation of their own goals, and the purpose of the facilitator is mainly to ensure that the goals formulated are all SMART goals (as outlined above).

##### Table 7: Action plan delivery self-rating checklist

| **Score** | **Quality level** | **Description of characteristics** |
| --- | --- | --- |
|  | 0 – Absence of skill | No skills were used in the formulation of the action plan. |
|  | 1 – Novice | The deliverer dictated most of the goals to the patient with little regard for the patient’s autonomy, and few of the goals meet SMART criteria. |
|  | 2 – Advanced beginner | The patient was partially supported in the goal making process. Some of the goals meet SMART criteria. Little evidence of a collaborative relationship between deliverer and patient. |
|  | 3 – Competent | The patient and deliverer had evidence of a collaborative relationship. Autonomy of the patient was mostly supportive and feedback was given for the majority of the goals as to whether they adhered to SMART criteria. |
|  | 4 – Proficient | Collaborative relationship between deliverer and patient was clear, and the patient confirmed that every goal adheres to SMART criteria. Autonomy of the patient was supported throughout the session. Small issues in delivery still present (e.g. couple opportunities missed to discuss an element of the SMART criteria). |
|  | 5 – Expert | Collaborative relationship between deliverer and patient was clear, and the patient confirmed that every goal adheres to SMART criteria. Autonomy of the patient was supported throughout the session. No discernible issues with delivery. |

##### Table 8: Action plan scoring table

| **Score** | **Quality level** | **Description of characteristics** |
| --- | --- | --- |
|  | 0 – Non-existent | There is no evidence for an action plan for this participant. |
|  | 1 – Inadequate | In total, fewer than 6 goals and/or environmental modifications are created. This could be 3 goals and 2 environmental modifications, for example. |
|  | 2 – Adequate | Only one or two items are missing in total. For example, only 5 goals and 2 environmental modifications may be present. Total score for the SMART criteria is above 8. |
|  | 3 – Good | All goals and environmental modifications are present and the total score is above 12 on the SMART criteria. |
|  | 4 – Very good | All goals and environmental modifications are present, and the goals have been rated and all of them adhere to at least 3 of the SMART criteria, plus a total score of 18 or above for total action plan. |
|  | 5 - Excellent | All goals are present, score of 25 or above for SMART criteria, and all are suitable for the physical function level of the participant. |

##### Table 9: Participant goal rating checklist

| Goal number | SMART criteria | | | | |  |
| --- | --- | --- | --- | --- | --- | --- |
|  | SPECIFIC | MEASURABLE | ACHIEVABLE | RELEVANT | TIMELY | Total score for goal (out of 5) |
| 1 |  |  |  |  |  |  |
| 2 |  |  |  |  |  |  |
| 3 |  |  |  |  |  |  |
| 4 |  |  |  |  |  |  |
| 5 |  |  |  |  |  |  |
| 6 |  |  |  |  |  |  |
| Total for action plan |  |  |  |  |  |  |

# References

Dreyfus, S. E. (2004) ‘The five-stage model of adult skill acquisition’, *Bulletin of Science, Technology and Society*, 24(3), pp. 177–181. doi: 10.1177/0270467604264992.
