# Supplementary material for: A novel behavioural INTErvention to REduce Sitting Time in older adults undergoing orthopaedic surgery (INTEREST): protocol for a randomised controlled feasibility study
Source: Pilot Feasibility Stud. 2019 Apr 6;5:54. doi: 10.1186/s40814-019-0437-2 (PMC6451782; doi:10.1186/s40814-019-0437-2)
Supplement: Supplementary file 4 — INTEREST Feasibility Questionnaire (control group). Feasibility questionnaire given to participants at visit 4 (pre-surgery) and visit 5 (post-surgery) to assess acceptability, adoption, practicality satisfaction, and safety of the study and to get additional feedback on study processes. (DOCX 22 kb) [file 40814_2019_437_MOESM4_ESM.docx]

| **Feasibility Questionnaire (Pre-Surgery)** |
| --- |

The following questionnaire relates to the study in which you have been taking part. It should take no more than ten minutes of your time. Please try to be as accurate as possible. Some questions require placing an X or a tick in the box of your choosing, others ask for a longer written answer. Your experiences and feedback are very important and will help inform the design of future research.

1. Have you found taking part in the study burdensome? *(Put a X in the appropriate box).*

| 1 | 2 | 3 | 4 | 5 |
| --- | --- | --- | --- | --- |
|  |  |  |  |  |
| Not  burdensome at all | | Somewhat burdensome | Very  burdensome | |

1a. If you found it burdensome, how could it have been improved?

1. Do you feel that taking part in the study has exposed you to more pain? *(Put a X in the appropriate box).*

| No pain | Some more pain | A lot more pain |
| --- | --- | --- |
|  |  |  |

1. Do you feel that taking part in the study has exposed you to risk of physical harm? *(Put a X in the appropriate box).*

| No risk | Some more risk | A lot more risk |
| --- | --- | --- |
|  |  |  |

3a. If you found an aspect of the study more painful or harmful than usual, please write it below:

1. How have you found the assessments in the study so far (e.g. the questionnaires, physical tests, etc.).
2. Is there anything you would change about these assessments? Please write them below:
3. How do you feel about being randomised into the group you are in in the study (control or intervention group)?

| 1 | 3 | 4 | 5 | 7 |
| --- | --- | --- | --- | --- |
|  |  |  |  |  |
| Very dissatisfied | Dissatisfied | Neither satisfied nor dissatisfied | Satisfied | Very satisfied |

1. How likely would you be to suggest taking part in such a study to friends or family? *(Put a X in the appropriate box).*

| Not likely | Quite unlikely | Neither likely nor unlikely | Quite likely | Very likely |
| --- | --- | --- | --- | --- |
|  |  |  |  |  |

1. How would you rate your overall satisfaction with the study? *(Put a X in the appropriate box).*

| 1 | 3 | 4 | 5 | 7 |
| --- | --- | --- | --- | --- |
|  |  |  |  |  |
| Very dissatisfied | Dissatisfied | Neither satisfied nor dissatisfied | Satisfied | Very satisfied |

1. Do you have any suggested improvements for the study? Please write them below.

Many thanks for your participation in this study.

| **Feasibility Questionnaire (Post-Surgery)** |
| --- |

The following questionnaire relates to how your participation in this study may have influenced your experiences after surgery. It should take no more than ten minutes of your time. Please try to be as accurate as possible. Some questions require placing an X or a tick in the box of your choosing, and others ask for a longer written answer.

1. Do you feel that taking part in the study has influenced your recovery after surgery? *(Put a X in the appropriate box).*

| Very negative impact | Somewhat negative impact | No impact | Somewhat positive impact | Positive impact |
| --- | --- | --- | --- | --- |
|  |  |  |  |  |

1. Have you found taking part in the study burdensome? *(Put a X in the appropriate box).*

| 1 | 2 | 3 | 4 | 5 |
| --- | --- | --- | --- | --- |
|  |  |  |  |  |
| Not  burdensome at all | | Somewhat burdensome | Very  burdensome | |

2a. If you found it burdensome, how could it have been improved?

1. Do you feel that taking part in the study has exposed you to more pain? *(Put a X in the appropriate box).*

| No pain | Some more pain | A lot more pain |
| --- | --- | --- |
|  |  |  |

1. Do you feel that taking part in the study has exposed you to risk of physical harm? *(Put a X in the appropriate box).*

| No risk | Some more risk | A lot more risk |
| --- | --- | --- |
|  |  |  |

4a. If you found an aspect of the study more painful or harmful than usual, please write it below:

1. How have you found the assessments in the study so far (e.g. the questionnaires, physical tests, etc.).
2. Is there anything you would change about these assessments? Please write them below:
3. How would you rate your overall satisfaction with the study? *(Put a X in the appropriate box).*

| 1 | 3 | 4 | 5 | 7 |
| --- | --- | --- | --- | --- |
|  |  |  |  |  |
| Very dissatisfied | Dissatisfied | Neither satisfied nor dissatisfied | Satisfied | Very satisfied |

1. How likely would you be to suggest taking part in such a study to friends or family? *(Put a X in the appropriate box).*

| Not likely | Quite unlikely | Neither likely nor unlikely | Quite likely | Very likely |
| --- | --- | --- | --- | --- |
|  |  |  |  |  |

1. Do you have any suggested improvements for the study? Please write them below.

Many thanks for your participation in this study.
