# Supplementary material for: A novel behavioural INTErvention to REduce Sitting Time in older adults undergoing orthopaedic surgery (INTEREST): protocol for a randomised controlled feasibility study
Source: Pilot Feasibility Stud. 2019 Apr 6;5:54. doi: 10.1186/s40814-019-0437-2 (PMC6451782; doi:10.1186/s40814-019-0437-2)
Supplement: Supplementary file 5 — INTEREST Feasibility Questionnaire (intervention group). Feasibility questionnaire given to participants at visit 4 (pre-surgery) and visit 5 (post-surgery) to assess acceptability, adoption, practicality satisfaction, and safety of the study and to get additional feedback on study processes. (DOCX 27 kb) [file 40814_2019_437_MOESM5_ESM.docx]

| **Feasibility Questionnaire (Pre-Surgery)** |
| --- |

The following questionnaire relates to the study in which you have been taking part. It should take no more than ten minutes of your time. Please try to be as accurate as possible. Some questions require placing an X or a tick in the box of your choosing, others ask for a longer written answer. Your experiences and feedback are very important and will help inform the design of future research.

1. Did you have any problems achieving the goals you set at the beginning of the study? *(Put a X in the appropriate box).*

| No problems | Some problems | Many problems |
| --- | --- | --- |
|  |  |  |

1. Did you have any problems achieving the environmental modifications? *(Put a X in the appropriate box).*

| No problems | Some problems | Many problems |
| --- | --- | --- |
|  |  |  |

1. How easy was it to achieve your goals physically? *(Put a X in the appropriate box).*

| Very difficult | Somewhat difficult | Neither difficult nor easy | Quite easy | Very easy |
| --- | --- | --- | --- | --- |
|  |  |  |  |  |

1. How easy was it to achieve your goals mentally? *(Put a X in the appropriate box).*

| Very difficult | Somewhat difficult | Neither difficult nor easy | Quite easy | Very easy |
| --- | --- | --- | --- | --- |
|  |  |  |  |  |

1. Did you find the goals to be well-suited to your individual circumstances? *(Put a X in the appropriate box).*

| Not well-suited at all | Quite not well-suited | Neither well-suited nor non-well-suited | Quite well-suited | Very well-suited |
| --- | --- | --- | --- | --- |
|  |  |  |  |  |

1. How useful did you find the goal booklet? *(Put a X in the appropriate box).*

| 1 | 2 | 3 | 4 | 5 |
| --- | --- | --- | --- | --- |
|  |  |  |  |  |
| Not  useful at all | |  | Very  useful | |

1. Do you have any suggested improvements for the booklet? Please write them below.
2. How do you feel about being randomised into the group you are in in the study (control or intervention group)?

| 1 | 3 | 4 | 5 | 7 |
| --- | --- | --- | --- | --- |
|  |  |  |  |  |
| Very dissatisfied | Dissatisfied | Neither satisfied nor dissatisfied | Satisfied | Very satisfied |

1. Have you found taking part in the study burdensome? *(Put a X in the appropriate box).*

| 1 | 2 | 3 | 4 | 5 |
| --- | --- | --- | --- | --- |
|  |  |  |  |  |
| Not  burdensome at all | | Somewhat burdensome | Very  burdensome | |

9a. If you found it burdensome, how could it have been improved?

1. Do you feel that taking part in the study has exposed you to more pain? *(Put a X in the appropriate box).*

| No pain | Some more pain | A lot more pain |
| --- | --- | --- |
|  |  |  |

1. Do you feel that taking part in the study has exposed you to risk of physical harm? *(Put a X in the appropriate box).*

| No risk | Some more risk | A lot more risk |
| --- | --- | --- |
|  |  |  |

11a. If you found an aspect of the study more painful or harmful than usual, please write it below:

1. Could you have changed your goals to make them more achievable?

| Yes | No |
| --- | --- |
|  |  |

12a. If yes, how could you have changed your goals to make them more achievable?

1. Which part(s) of the intervention did you find most difficult?
2. Which part(s) of the intervention did you find most enjoyable?
3. How would you rate your overall satisfaction with the study? *(Put a X in the appropriate box).*

| 1 | 3 | 4 | 5 | 7 |
| --- | --- | --- | --- | --- |
|  |  |  |  |  |
| Very dissatisfied | Dissatisfied | Neither satisfied nor dissatisfied | Satisfied | Very satisfied |

1. How likely would you be to suggest taking part in such a study to friends or family? *(Put a X in the appropriate box).*

| Not likely | Quite unlikely | Neither likely nor unlikely | Quite likely | Very likely |
| --- | --- | --- | --- | --- |
|  |  |  |  |  |

1. How likely are you to continue working towards your goals in the future? *(Put a X in the appropriate box).*

| Not likely | Quite unlikely | Neither likely nor unlikely | Quite likely | Very likely |
| --- | --- | --- | --- | --- |
|  |  |  |  |  |

1. Do you have any suggested improvements for the study? Please write them below.

| **Feasibility Questionnaire (Post-Surgery)** |
| --- |

1. Since you had your surgery, have you been working towards achieving the goals set in the study? *(Put a X in the appropriate box).*

| Not at all | A little | Very much so |
| --- | --- | --- |
|  |  |  |

1. How difficult has it been to work towards the goals set earlier in the study in the absence of the phone calls, etc.? *(Put a X in the appropriate box).*

| Very difficult | Somewhat difficult | Neither difficult nor easy | Quite easy | Very easy |
| --- | --- | --- | --- | --- |
|  |  |  |  |  |

1. Could your goals have been easier to achieve since surgery?

| Yes | No |
| --- | --- |
|  |  |

3a. If so, do you have any ideas how?

1. How have you found the assessments in the study so far (e.g. the questionnaires, physical tests, etc.)?
2. Do you feel that taking part in the study has influenced your recovery after surgery? *(Put a X in the appropriate box).*

| Very negative impact | Somewhat negative impact | No impact | Somewhat positive impact | Positive impact |
| --- | --- | --- | --- | --- |
|  |  |  |  |  |

1. Have you continued to achieve your environmental modifications since surgery? *(Put a X in the appropriate box).*

| None of them | One or two | All of them |
| --- | --- | --- |
|  |  |  |

1. How likely are you to continue working towards your goals in the future? *(Put a X in the appropriate box).*

| Very unlikely | Quite unlikely | Neither likely nor unlikely | Quite likely | Very likely |
| --- | --- | --- | --- | --- |
|  |  |  |  |  |

1. Do you see yourself continuing to achieve the environmental modifications in the future? *(Put a X in the appropriate box).*

| None of them | One or two | All of them |
| --- | --- | --- |
|  |  |  |

1. Have you continued to use the sedentary behaviour booklet since surgery? *(Put a X in the appropriate box).*

| Not at all | A little | Very much so |
| --- | --- | --- |
|  |  |  |

1. Will you continue to use the sedentary behaviour booklet in the future? *(Put a X in the appropriate box).*

| Not at all | A little | Very much so |
| --- | --- | --- |
|  |  |  |

1. How would you rate your overall satisfaction with the study? *(Put a X in the appropriate box).*

| 1 | 3 | 4 | 5 | 7 |
| --- | --- | --- | --- | --- |
|  |  |  |  |  |
| Very dissatisfied | Dissatisfied | Neither satisfied nor dissatisfied | Satisfied | Very satisfied |

1. How likely would you be to suggest taking part in such a study to friends or family? *(Put a X in the appropriate box).*

| Not likely | Quite unlikely | Neither likely nor unlikely | Quite likely | Very likely |
| --- | --- | --- | --- | --- |
|  |  |  |  |  |

1. Do you have any suggested improvements for the study? Please write them below.
2. Have you found taking part in the study burdensome? *(Put a X in the appropriate box).*

| 1 | 2 | 3 | 4 | 5 |
| --- | --- | --- | --- | --- |
|  |  |  |  |  |
| Not  burdensome at all | | Somewhat burdensome | Very  burdensome | |

14a. If you found it burdensome, how could it have been improved?

Many thanks for your participation in this study.
