# Supplementary material for: A novel behavioural INTErvention to REduce Sitting Time in older adults undergoing orthopaedic surgery (INTEREST): protocol for a randomised controlled feasibility study
Source: Pilot Feasibility Stud. 2019 Apr 6;5:54. doi: 10.1186/s40814-019-0437-2 (PMC6451782; doi:10.1186/s40814-019-0437-2)
Supplement: Supplementary file 6 — Topic guide for the interview with research nurses. Topic guide to provide information about what the interview regarding feasibility of recruitment with research nurses entails. (DOCX 16 kb) [file 40814_2019_437_MOESM6_ESM.docx]

1. Do you feel that the recruitment strategy in INTEREST was effective?
2. Did you encounter any major roadblocks to recruitment?
3. If you mentioned any issues, how could these have been addressed?
4. Do you think that it would be possible to have greater integration between researchers and hospital staff for similar studies in the future?
5. In your opinion, could any of the study documents have been better designed to streamline recruitment?

6. Were there any procedures you were carrying out that could have been more streamlined, and if so, how?
7. Were there any other avenues other than the ones used in INTEREST that could have enhanced recruitment (e.g. clinic posters, etc.)?
8. Could you foresee any issues if a new version of INTEREST were to run at another research site (i.e. another hospital)?
9. Which times of year were/are most difficult for recruitment?
10. At what point would more RNs be needed to be able to send PIS' to all eligible patients (e.g. would it be at 40/mth. 100/mth, etc.)?
11. How many PIS' were sent per month on average?
12. Did your workload ever mean that you had to prioritise studies for recruitment, and if so, did this influence recruitment to INTEREST?
13. Did you manage to telephone all participants 1-week after sending the PIS? If not, what could have made this easier to achieve?

*This document reflects the questions that will be asked to the research nurses during the feasibility interview for INTEREST. However, due to its semi-structured nature, it may be possible that slight divergences will occur to further explore in detail any nuances that arise during the process. All discussions will stick to the topic of recruitment in the INTEREST study.*
