# Supplementary material for: A novel behavioural INTErvention to REduce Sitting Time in older adults undergoing orthopaedic surgery (INTEREST): protocol for a randomised controlled feasibility study
Source: Pilot Feasibility Stud. 2019 Apr 6;5:54. doi: 10.1186/s40814-019-0437-2 (PMC6451782; doi:10.1186/s40814-019-0437-2)
Supplement: Supplementary file 7 — Rationale for exploratory outcomes in the INTEREST feasibility study. (DOCX 47 kb) [file 40814_2019_437_MOESM7_ESM.docx]

Rationale for exploratory outcome measures in the INTEREST feasibility study

## Objective measurement of movement and sedentary behaviour (ActivPal)

The ActivPal developed by PAL Technologies, Glasgow, United Kingdom, is an activity monitor that is well-validated and highly suited to measuring sedentary behaviour variables, such as sitting time and sit-to-stand transitions [1, 2]. It was chosen as the activity monitor of choice because of its inclinometer and sedentary behaviour monitoring functions and will be used at multiple timepoints to assess a number of variables. Due to the ability to measure both posture and movement, it is possible to extract data relating to sedentariness, including sitting time, physical activity, average length of sedentary bouts, sit-to-stand transitions, and the number of sedentary bouts above a certain length. It can also capture stepping time (walking) but cannot determine the intensity of a physical activity being performed. The ActivPal is an objective measurement tool of sedentary behaviour/activity, and thus cannot determine what behaviours are being performed at specific times or which are being reduced. For that reason, a self-report method should be used in conjunction with it. However, even though it cannot determine the context of behaviours, the ActivPal is unmatched in its accuracy of measurement of sedentariness and activity when compared with direct observation, with 100% accuracy for standing, and over 95% accuracy for stepping and sitting behaviours, and correct detection of cycling as activity 93% of the time in healthy adults [3].

## Physical function - Short Physical Performance Battery (SPPB)

The SPPB is a multicomponent physical function test that assesses gait speed, balance, and lower limb power and strength with a possible score between 0 and 12, where 12 is maximal [4]. It has been found to be sensitive to events such as hospitalisation and rehabilitation and thus is an appropriate measure of change in physical performance in older adults undergoing surgery [5, 6]. The SPPB has also been effective at predicting which individuals are likely to lose their ability to pass a 400m walk test, which makes it a useful tool for identifying individuals most at need for intervention [7]. In a previous study of a 12-week intervention to reduce sitting in older adults, the SPPB was found to improve by 0.5 points, mostly due to increases in the chair stand portion of the test [8]. A 0.5 point increase is likely to be indicative of a significant improvement to wellbeing in many individuals, as a secondary analysis of observational data and clinical trials in 692 people has shown that an increase in score of 0.5 is a useful target indicative of a clinically meaningful difference [9]. For these reasons, it was used at all three assessment points of the study to assess whether a change in physical function had occurred.

## Self-reported physical activity - International Physical Activity Questionnaire Short Form (IPAQ-SF)

The IPAQ-SF assesses physical activity behaviours through self-report methods. The IPAQ-SF has been found to be lacking in validity in comparison to objective measures of activity in a recent systematic review which included n=23 validation studies, overestimating by an average of 84 percent [10]. Only vigorous physical activity and walking were found to have acceptable validity [10]. Validity and reliability are also lacking for assessment of sedentary behaviours [11]. For this reason, the Measure of Older Adults Sedentary Time (MOST) will be used in INTEREST, in addition to the IPAQ-SF, to determine which sedentary behaviours were performed in which contexts. However, the IPAQ-SF nonetheless provided valuable data about the quantity of light, moderate, and vigorous physical activity undertaken by participants. A study comparing n=225 chronic musculoskeletal disorder patients with n=350 control subjects using the IPAQ-SF found that these two groups differed mostly on vigorous physical activity (VPA), as the patient group reported 0 MET/hours of VPA per week, compared to 240 for the controls [12]. The other reported indices of physical activity were not significantly different, showing that physical function or disease limitations may differentially affect subtypes of physical activity, and thus it is important to capture these separately.

## Self-reported sedentary time - Measure of Older Adults’ Sedentary Time (MOST)

The measure of Older Adults’ Sedentary Time was developed specifically for the measurement of older adult’s sedentary time, offering a number of domain-specific measures of behaviour, such as reading, watching television, or doing hobbies, and has been validated in older adults [13]. It has good test-retest reliability on most subdomains, with a score of 0.78 for TV time, but less so for socialising and transport, and was acceptable for overall sedentary time (0.52). However, in this study, objective measurement was used alongside the MOST, thus providing both accurate measurement of overall activity and sedentariness, whilst using the MOST to acquire contextual information. The MOST has been used in a recent sedentary behaviour intervention in older adults, and was found to consistently be more accurate for reporting overall sitting time than the IPAQ, which typically underreported [14].

## Nutritional status - Short-Form Mini Nutritional Assessment (SF-MNA)

The SF-MNA was specifically developed to assess whether older adults are eating a sufficiently nutritious diet, and has correlation of 0.94 with the full Mini Nutritional Assessment tool [15]. It was included in this study as an effective tool for assessing malnutrition in participants, enabling malnutrition to be controlled for. For example, if a participant is found to have a particularly low physical function (SPPB) score, and is also found to be malnourished, then it would be possible for malnutrition to be a confound with respect to the results of the study. Nutritional status can therefore be used as a covariate to control for nutritional status when performing analyses if required.

## Activities of daily living - Katz Index of Independence in Activities of Daily Living (Katz ADL)

Developed in 1979, the Katz ADL was developed specifically to address the growing need for a tool which can assess functional capacity for everyday tasks in older adults [16]. The Katz ADL asks a number of questions of the older individual, namely whether they can perform several daily tasks on their own, such as bathing, dressing, toileting, and feeding. It is scored from 0-6, with 6 being most independent. In this study, the Katz ADL will be used to assess whether individuals change in terms of dependence over the course of the study, particularly as a result of their surgery. Katz ADL has even been found to be sensitive to change as a result of interventions. One year-long intervention in n=134 patients in nursing homes with Alzheimer’s disease that used two weekly, 1 hour exercise sessions, found that the patients had significantly less decline in independence over the 1-year period in the exercise group [17].

## Basic Psychological Needs in general Scale (BPNS)

This intervention was designed using the principles of Self-Determination Theory (SDT). Thus, it was important to assess whether or not some of the constructs within the theory change as a result of taking part in the intervention, namely the Basic Psychological Needs of Autonomy, Relatedness, and Competence. The BPNS questionnaire includes 7 items per psychological need, for a total of 21 items. Satisfaction of the three basic needs has been shown to be associated with positive well-being, life satisfaction, self-esteem, and negatively linked to depression [18]. In the INTEREST study, this measure was incorporated to identify whether participants in the study felt like their basic psychological needs had been supported or enhanced by working towards their self-formulated goals, rather than being extrinsically mandated. By measuring the basic psychological needs, it would be possible to measure whether the individuals in the study feel that their competence, autonomy, and relatedness were enhanced as a result of the intervention.

## Quality of life - EuroQol-5D-5L (EQ-5D-5L) and EQ-VAS

The EQ-5D-5L is a measure of QoL and overall health, and includes five dimensions: mobility, self-care, usual activities, pain/discomfort and anxiety/depression. Each dimension has 5 possible degrees of QoL: no problems, slight problems, moderate problems, severe problems and extreme problems. The EQ-5D-5L is regularly used in orthopaedic care in the UK, and is a common tool in research [19]. A review of instruments for assessing quality of life in older adults has emphasised that since QoL is a multi-dimensional concept, there is no one tool that yet adequately measures all aspects, but the EQ-5D-5L is found to be the best for assessing quality-adjusted life years [20]. In addition to the EQ-5D-5L, the EuroQol Visual Analogue Scale (EQ-VAS) was used, which allows for the participant to rate their overall health with a number from 0-100, where 0 is the worst health that they can imagine, and 100 the greatest. This enabled data collection about participants’ changing overall wellbeing during the study.

Hip and knee pain and recovery - Oxford Hip/Knee Score
The Oxford Hip and Knee scores have been in use since the late 1990s, and today are routinely used in orthopaedic care to judge the success of hip and knee replacements [21, 22]. This assessment is delivered in the form of a self-completed questionnaire delivered to the patient, which helps reduce sources of bias by avoiding the or interpretation of the clinician [23]. The questions asked are on a 1-5 scale and mostly relate to pain or obstructions to daily live caused by the hip or knee issues, such as “could you do household shopping on your own?”, and, “how much has pain interfered with your usual work (including housework)?”. Both are scored from 0-60, where 60 is perfect hip or knee function without any pain. Other studies have found that a change in score of 5 points is indicative of an actual improvement to the impact of a hip or knee condition on one’s life, rather than being attributable to measurement error [24]. Thus, by comparing the control and intervention group in addition to pre vs. post data, it would be possible to judge the magnitude of any benefit to the participant in terms of their perception of the impact of their knee/hip condition on their lives. It will also aid in assessing changes in both groups across all three timepoints.

## Cardiometabolic health - Blood Measures

One of the most unique aspects of this study is the assessment of cardiometabolic biomarkers. To date, no study in older adults has assessed the impact on cardiometabolic biomarkers of a sitting time reduction [25]. It is not yet clear whether a reduction in sitting time is potent enough to meaningfully change these markers. This intervention includes blood sampling at baseline and pre-surgery timepoints for analysis of Albumin, HDL, LDL, triglycerides, Vit. D., CRP, Cortisol, DHEA/S, and Transferrin. These measures have been chosen due to their strong associations with cardiovascular disease processes [26].

## Waist to Hip Ratio (WHR)

Many studies measure waist circumference (WC) as a means of assessing overweight, where it has been shown to be more effective possibly due to accounting for abdominal adiposity in particular (the most dangerous form of fat distribution) [27]. In terms of CVD risk, a meta-regression analysis of 15 articles with over 250,000 participants found that WHR better predicts CVD events than WC alone does [28]. This may be because it considers relative bone structure, which can better expose actual adiposity. Of course, the best measure would still be DXA scanning, but unfortunately this study does not have the resources for this. In a study following 14,833 older adults over the age of 75, recruited from family practices in the UK, WHR has been found to be more strongly associated with overall mortality than BMI or WC [29]. There was no association found between WC and mortality [29]. In relation to osteoarthritis, a population-based cohort study of Swedish individuals found that there is an increasing relative risk (RR) for OA with increases in BMI, WC, weight, body fat percentage, and WHR [30]. Interestingly, BMI was found to be most predictive of osteoarthritis, with a RR=8.1 for 1^st^ vs. 4^th^ quartile of BMI with knee OA, and 2.6 for BMI [30]. However, since the patients in this study already have OA, using WHR will likely give better insight into whether the study could have a beneficial effect on cardiometabolic health than using BMI alone. Given the benefits of WHR over WC, and the small amount of extra time required to do the extra hip measurement, there was no reason not to choose measurement of WHR for this study.

# References

1. Ryan CG, Grant PM, Tigbe WW, Granat MH, Ryan CG. The validity and reliability of a novel activity monitor as a measure of walking. Br J Sport Med. 2006;40 table 1:779–84.

2. Lyden K, Kozey Keadle SL, Staudenmayer JW, Freedson PS. Validity of two wearable monitors to estimate breaks from sedentary time. Med Sci Sports Exerc. 2012;44:2243–52.

3. Steeves JA, Bowles HR, McClain JJ, Dodd KW, Brychta RJ, Wang J, et al. Ability of thigh-worn actigraph and activpal monitors to classify posture and motion. Med Sci Sports Exerc. 2015;47:952–9.

4. Guralnik JM, Simonsick EM, Ferrucci L, Glynn RJ, Berkman LF, Blazer DG, et al. A short physical performance battery assessing lower extremity function: association with self-reported disability and prediction of mortality and nursing home admission. J Gerontol. 1994;49:M85-94. doi:10.1093/geronj/49.2.M85.

5. Volpato S, Cavalieri M, Sioulis F, Guerra G, Maraldi C, Zuliani G, et al. Predictive Value of the Short Physical Performance Battery Following Hospitalization in Older Patients. Journals Gerontol Ser A. 2010;66A:89–96. http://dx.doi.org/10.1093/gerona/glq167.

6. Bunout D, Barrera G, Leiva L, Gattas V, de la Maza MP, Avendaño M, et al. Effects of vitamin D supplementation and exercise training on physical performance in Chilean vitamin D deficient elderly subjects. Exp Gerontol. 2006;41:746–52. doi:10.1016/j.exger.2006.05.001.

7. Vasunilashorn S, Coppin AK, Patel K V, Lauretani F, Ferrucci L, Bandinelli S, et al. Use of the Short Physical Performance Battery Score to Predict Loss of Ability to Walk 400 Meters : Analysis From the InCHIANTI Study. 2009;64:223–9.

8. Gibbs BB, Brach JS, Byard T, Creasy S, Davis KK, McCoy S, et al. Reducing Sedentary Behavior Versus Increasing Moderate-to-Vigorous Intensity Physical Activity in Older Adults: A 12-Week Randomized, Clinical Trial. J Aging Health. 2016.

9. Perera S, Mody ÃSH, Woodman RC, Studenski SA, Mph Ã. Meaningful Change and Responsiveness in Common Physical Performance Measures in Older Adults. 2006;:743–9.

10. Lee PH, Macfarlane DJ, Lam TH, Stewart SM. Validity of the international physical activity questionnaire short form ( IPAQ-SF ): A systematic review. Int J Behav Nutr Phys Act. 2011;8:1–11.

11. Rosenberg DE, Bull FC, Marshall AL, Sallis JF, Bauman AE. Assessment of Sedentary Behavior with the International Physical Activity Questionnaire. J Phys Act Heal. 2008;5:S30–44. doi:10.1123/jpah.5.s1.s30.

12. Moseng T, Tveter AT, Holm I, Dagfinrud H. Patients with musculoskeletal conditions do less vigorous physical activity and have poorer physical fitness than population controls: A cross-sectional study. Physiother (United Kingdom). 2014;100:319–24. doi:10.1016/j.physio.2013.11.005.

13. Gardiner PA, Clark BK, Healy GN, Eakin EG, Winkler EAH, Owen N. Measuring Older Adults’ Sedentary Time: Reliability, Validity, and Responsiveness. Med Sci Sport Exerc. 2011;43:2127–33. doi:10.1249/MSS.0b013e31821b94f7.

14. White I, Smith L, Aggio D, Shankar S, Begum S, Matei R, et al. On Your Feet to Earn Your Seat: pilot RCT of a theory-based sedentary behaviour reduction intervention for older adults. Pilot Feasibility Stud. 2017;3:23. doi:10.1186/s40814-017-0139-6.

15. Rubenstein LZ, Harker JO, Salvà A, Guigoz Y, Vellas B. Screening for Undernutrition in Geriatric Practice: Developing the Short-Form Mini-Nutritional Assessment (MNA-SF). J Gerontol Med Sci Public Domain. 2001;56:366–72.

16. Katz S. Assessing Self-maintenance : Activities of Daily Living , Mobility , and Instrumental Activities. 1979;:721–7.

17. Rolland Y, Pillard F, Klapouszczak A, Reynish E, Thomas D, Andrieu S, et al. Exercise Program for Nursing Home Residents with Alzheimer’s Disease: A 1-Year Randomized, Controlled Trial. J Am Geriatr Soc. 2007;55:158–65. doi:10.1111/j.1532-5415.2007.01035.x.

18. Johnston MM, Finney SJ. Measuring basic needs satisfaction : Evaluating previous research and conducting new psychometric evaluations of the Basic Needs Satisfaction in General Scale. Contemp Educ Psychol. 2010;35:280–96. doi:10.1016/j.cedpsych.2010.04.003.

19. Ng JYY, Ntoumanis N, Thøgersen-Ntoumani C, Deci EL, Ryan RM, Duda JL, et al. Self-Determination Theory Applied to Health Contexts: A Meta-Analysis. Perspect Psychol Sci. 2012.

20. Bulamu NB, Kaambwa B, Ratcliffe J. A systematic review of instruments for measuring outcomes in economic evaluation within aged care. Health Qual Life Outcomes. 2015;13:1–23.

21. Dawson J, Fitzpatrick R, Murray D, Carr A. Questionnaire on the perceptions of patients about total knee replacement. 1998;80 January:63–9.

22. Dawson J, Fitzpatrick R, Carr A. Questionnaire on the perceptions of patients about total hip replacement. J Bone Jt Surg. 1996;78 March:185–90.

23. Murray DW, Fitzpatrick R, Rogers K, Pandit H, Beard DJ, Carr AJ, et al. The use of the Oxford hip and knee scores. J Bone Jt Surg. 2007;:1010–4.

24. Beard DJ, Harris K, Dawson J, Doll H, Murray DW, Carr AJ, et al. Meaningful changes for the Oxford hip and knee scores after joint replacement surgery. J Clin Epidemiol. 2015;68:73–9. doi:10.1016/j.jclinepi.2014.08.009.

25. Aunger JA, Doody P, Greig CA. Interventions targeting sedentary behavior in non-working older adults: a systematic review. Maturitas. 2018. doi:https://doi.org/10.1016/j.maturitas.2018.08.002.

26. Duarte MMMF, Rocha JBT, Moresco RN, Duarte T, Da Cruz IBM, Loro VL, et al. Association between ischemia-modified albumin, lipids and inflammation biomarkers in patients with hypercholesterolemia. Clin Biochem. 2009;42:666–71. doi:10.1016/j.clinbiochem.2009.01.010.

27. Janssen I, Katzmarzyk PT, Ross R. Waist circumference and not body mass index explains obesity- related health risk 1 – 3. Am J Clin Nutr. 2004;79 March:379–84.

28. De Koning L, Merchant AT, Pogue J, Anand SS. Waist circumference and waist-to-hip ratio as predictors of cardiovascular events: Meta-regression analysis of prospective studies. Eur Heart J. 2007;28:850–6.

29. Price GM, Uauy R, Breeze E, Bulpitt CJ, Fletcher AE. Weight, shape, and mortality risk in older persons: Elevated waist-hip ratio, not high body mass index, is associated with a greater risk of death. Am J Clin Nutr. 2006;84:449–60.

30. Lohmander LS, De Verdier MG, Rollof J, Nilsson PM, Engström G. Incidence of severe knee and hip osteoarthritis in relation to different measures of body mass: A population-based prospective cohort study. Ann Rheum Dis. 2009;68:490–6.
