# Supplementary material for: A novel behavioural INTErvention to REduce Sitting Time in older adults undergoing orthopaedic surgery (INTEREST): protocol for a randomised controlled feasibility study
Source: Pilot Feasibility Stud. 2019 Apr 6;5:54. doi: 10.1186/s40814-019-0437-2 (PMC6451782; doi:10.1186/s40814-019-0437-2)
Supplement: Supplementary file 8 — Participant informed consent form for the INTEREST study. (DOCX 127 kb) [file 40814_2019_437_MOESM8_ESM.docx]

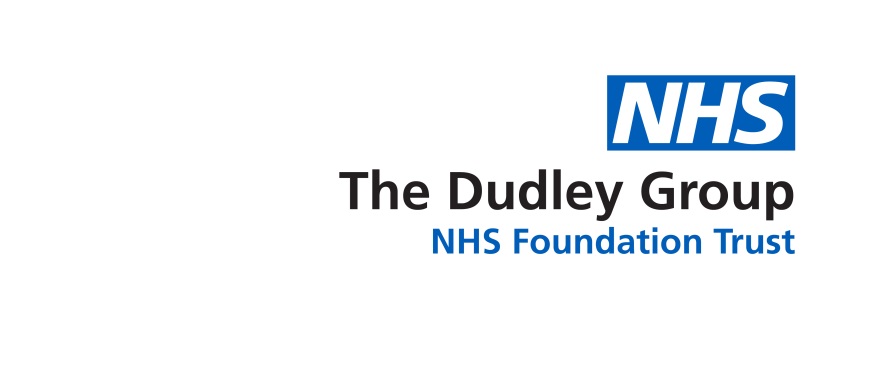

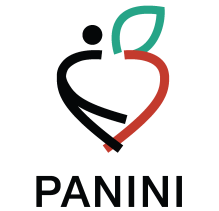

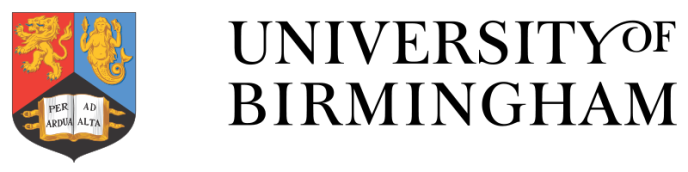

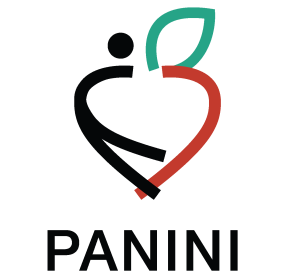
**Participant Informed Consent Form**

**Title of Project:** Reducing sitting time in older adults undergoing orthopaedic surgery: a feasibility study (INTEREST)

**Researchers:** Mr. Justin Aunger, Dr. Carolyn Greig, Prof. Ed Davis, Prof. Colin Greaves

Participant ID:

Please read each of the statements and decide whether you agree. To agree to an item, please sign your initials in the box. If you do not agree then please leave the box blank. All non-optional items must be initialled if you want to take part in the study.

| 1 | I confirm that I have read and understood the contents of the “Reducing sitting time in older adults before orthopaedic surgery: a feasibility study (INTEREST)” Participant Information Sheet version 4.1 dated 31.07.18 |  |
| --- | --- | --- |
| 2 | I agree that I have had the opportunity to ask the research team questions about my participation in the study and I am satisfied with the answers |  |
| 3 | I agree to being called on my personal telephone during the study by a member of the study team |  |
| 4 | I agree to my contact details being stored at the School of Sport, Exercise, and Rehabilitation Sciences at the University of Birmingham |  |
| 5 | I understand that data collected about me will be stored on a computer at the School of Sport, Exercise, and Rehabilitation Sciences at the University of Birmingham and will be link-anonymised so that I cannot be identified |  |
| 6 | I understand that relevant sections of data collected during the study may be looked at by regulatory authorities or appropriate individuals from the University of Birmingham, where it is relevant to me taking part in this research. I give permission for these individuals to have access to my data |  |
| 7 | I understand that this consent form and the master sheet (linking my name to my ID number within the study) will be stored in a locked filing cabinet, separate from all other data collected about me, in a locked data storage room in the School of Sport, Exercise and Rehabilitation Sciences at the University of Birmingham |  |
| 8 | I agree to blood samples being taken and stored at the University of Birmingham and I understand that my sample/data will be completely anonymised and that I cannot be identified from the sample (optional) |  |
| 9 | I agree to blood samples being transferred to a PANINI project partner, the University of Bologna, for epigenetic analysis (optional) |  |
| 10 | I agree to data collected in this study being shared with partner European institutions as part of the PANINI project shared dataset and that this data will be fully anonymised (optional) |  |
| 11 | I agree to having the audio of the discussion in visit 2 recorded if I am allocated to the sitting time reduction group. This audio will be kept anonymous and used only for quality checking purposes (optional) |  |
| 12 | I agree with data being shared with collaborating institutions, NHS Trusts, Universities and Commercial partners within and outside of the UK now and in the future. I understand that this data will be fully anonymised and cannot be used to identify me |  |
| 13 | I understand that I can withdraw from the study, at any time, without having to give a reason, and that any data collected up until that point will still be used |  |
| 14 | I consent to my data being stored for use in ethically approved research in the future |  |
| 15 | I agree to my GP being informed of my participation in this study |  |
| 16 | I agree to participate in this study |  |

Name of Participant Date Participant Signature

__________________ ______ ________________ _____________________

Name of Researcher Date Researcher Signature

________________________ _________________ _____________________

Thank you for your consideration of this study
