# Supplementary material for: A novel behavioural INTErvention to REduce Sitting Time in older adults undergoing orthopaedic surgery (INTEREST): protocol for a randomised controlled feasibility study
Source: Pilot Feasibility Stud. 2019 Apr 6;5:54. doi: 10.1186/s40814-019-0437-2 (PMC6451782; doi:10.1186/s40814-019-0437-2)
Supplement: Supplementary file 9 — Participant information sheet for the INTEREST study. (DOCX 371 kb) [file 40814_2019_437_MOESM9_ESM.docx]

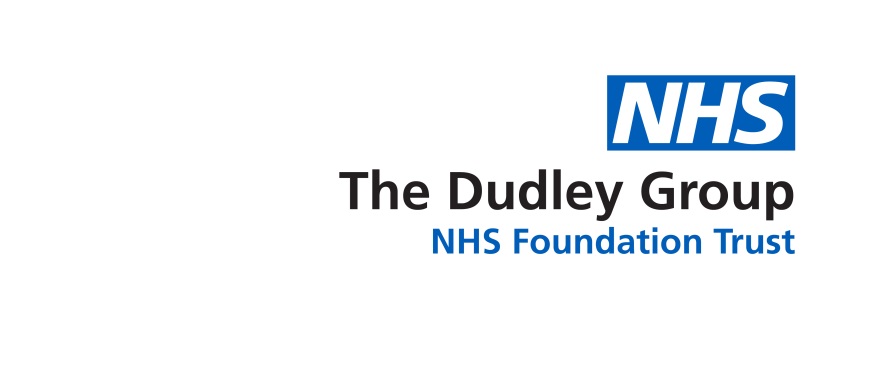

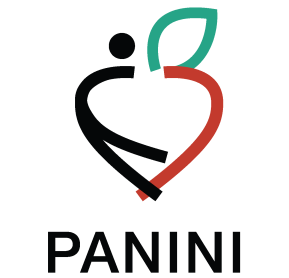
**
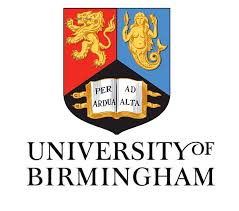
**

Russells Hall Hospital

Dudley

West Midlands

DY1 2HQ

# Participant Information Sheet

Reducing sitting time in older adults undergoing orthopaedic surgery: a feasibility study (INTEREST)

**Researchers:**

Mr. Justin Aunger; Dr. Carolyn Greig; Prof. Ed Davis, Prof. Colin Greaves

We would like to invite you to take part in our study. Before you decide, we’d like you to understand why this study is being done and what it would involve for you. Please take time to read the following information carefully. Feel free to ask questions using the contact info at the end if anything you read is not clear or you would like more information. Take your time to decide whether you wish to take part.

# What is the purpose of the study?

The purpose of the study is to help people waiting for hip and knee replacement surgeries to be more active before their surgery in a way that fits into their own lifestyle, regardless of their current activity level. Evidence suggests that reducing the amount of time spent sitting can increase health and physical abilities, but we do not know exactly how much of a reduction is needed to achieve this goal. This study aims to assess whether it is possible to decrease sitting time in older adults in the weeks before and after hip or knee surgery. One of our other objectives is to look at the effects on your health and how well you can move. To make sure we know if it is possible to reduce the time you spend sitting, a computer will randomly place you into one of two groups. One group will receive usual orthopaedic pre-operative care, and the other group will try to reduce the amount of time they spend sitting. Participants in both groups will have three points where measurements will be taken.

**Why have I been invited?**

You have been invited to take part in this research because you have been scheduled for hip or knee surgery at Russells Hall Hospital, are 65 years or older, and have access to a phone. We would like individuals of all activity levels to take part, even if you are active already.

**Do I have to take part?**

The decision to take part is entirely up to you. Once you have read the information sheet and your questions have been answered and you decide to take part, then the next step is to read and sign a consent form, a copy of which you can keep. You are free to withdraw from the study at any time, without having to give a reason and your medical care will be unaffected. If you are worried about leaving the home or having to travel in order to take part, the entire study can take place at your own home as the researcher can come to you for all the study visits.

**What will happen to me if I take part?**

This study will last for approximately 15-18 weeks, during which you will mostly go about your day to day activities as normal. If you are assigned to the group which is aiming to reduce sitting time, you will have four or five visits with the researcher, and one of these may be with other participants in the study. If you are in the usual care group, you will have only three visits (visits 1, 4, and 5). The visits are detailed as follows:

**Visit 1 – all participants**

To get started with the study, you will meet a member of our study team at either your own home, Russells Hall Hospital, the School of Sport, Exercise and Rehabilitation Sciences at the University of Birmingham. If you choose the University of Birmingham or Russells Hall Hospital, you will have your transport costs reimbursed by the study budget. At this meeting, you can read and sign the consent form indicating your willingness to take part. Once the consent form is signed, a few assessments will take place. Prior to this meeting you will need to wait to have breakfast until after the meeting as the blood measurements must be taken in a fasted state. These blood samples, used to assess your health, are equal to approximately 2 tablespoons of blood, and will be taken either by a trained member of the study team or by a nurse. During this visit, physical tests, a few short questionnaires about physical activity and food intake, and some physical measurements will be made. This session should take approximately one and a half hours, and if it takes place at either Russells Hall Hospital or the University of Birmingham, some breakfast will be provided for you. When this session ends, you will be asked to wear a physical activity monitor for at least three full days in a row. A return stamp-addressed envelope will also be provided. Once the three days is over, you should place the monitor back into the stamped and addressed envelope and post it back to the research team.

**Visit 2 – Sitting time reduction group only**

Shortly after the first session, we will invite you to take part in an informal group discussion. This discussion may either be in a group with other participants and a member of the study team, or individually with the researcher. This depends on scheduling and your own personal preference, and if it takes place individually, then visit 2 and visit 3 are combined into one visit. This meeting can either take place at your own home, Russells Hall Hospital, or the School of Sport, Exercise and Rehabilitation Sciences at the University of Birmingham, and should last around 60 minutes. This will give you the opportunity to think about and discuss your own sitting behaviours with other people who have similar experiences, and to discuss how you can go about changing these behaviours. During the session, a member of the study team will also provide education about the health impact of sitting for prolonged periods of time. At the end of the session, we hope you will be more aware of how you can reduce your sitting throughout your daily life. Additionally, we would like to audio record some of this session so that other members of the study team can make sure that this part of the study is being delivered to a high standard. However, this recording is completely optional and will not be linked to your personal information. The audio recording will stay on the audio recording device and will not be transferred onto any computer, and will only be accessed by members of the study team. As soon as the study team have listened to the recording, it will be destroyed.

**Visit 3 – Sitting time reduction group only**

Once this session is finished, you will also be booked into a session with a member of the study team, during which we will agree upon six goals to work towards with the aim of reducing your sitting time. This can take place at either Russells Hall Hospital, the School of Sport, Exercise and Rehabilitation Sciences at the University of Birmingham, or your home as per your preference. Your transport or parking costs will be reimbursed from the study budget. Additionally, in this session, we will agree upon three ways of modifying your home environment to make sitting down for lengthy periods less likely to occur. An example of a home modification could be simply moving the TV remote further away so that you have to get up to change the channel, or setting out your outdoor clothes at night so you can put them on in the morning, ready to go for a walk. Every other week, you will also receive a phone call from a member of the study team in which your progress will be discussed, and tips and encouragement will be offered to enhance your ability to reach your goals. Between each visit, you will be working towards these goals on your own at home. This visit should take between 60 and 90 minutes.

**Visit 4 – all participants**

Between 5-10 weeks after the first visit, you will have another visit with a member of the study team – either at your own home, the School of Sport, Exercise and Rehabilitation Sciences at the University of Birmingham, or at Russells Hall Hospital, whichever you prefer. This visit will be organised around your surgery date and should take place in the week before your surgery. At this time, you will repeat some of the tests that occurred during the first visit, including the optional blood samples (equal to about two tablespoons), so you will need to postpone your breakfast again, as the samples must be taken in a fasted state. It is possible that these blood samples may be taken by a member of your direct care team prior to your surgery to avoid you having to have an extra blood sampling session. Additionally, physical tests, short questionnaires, and physical measurements will be done. This session should take about one and a half hours. At the end of this session, you will again be asked to wear the physical activity monitor for at least three full days, after which you should return it in the envelope provided. For some people, this visit might be difficult to schedule, or be too close to their surgery and therefore too much to ask. In this case, we can do a reduced amount of the measures or do a reduced amount of assessments just over the phone. Please just indicate your preferences to the researcher. Shortly after this visit, you should have your surgery.

**Visit 5 – all participants**

The final visit is six weeks after your surgery to see how well your recovery is going. This meeting may take place at either your own home, the University of Birmingham, or at Russells Hall Hospital according to your preference. Your transport or parking costs will be reimbursed. This visit will include a reduced number of assessments (mostly questionnaires), and will last for approximately one hour. You will also be asked to wear the physical activity monitor again once more for 3-7 days, after which it should be returned to the study team in the envelope as before.

| **Visit Number** | **Group** | |
| --- | --- | --- |
|  | **Sitting Time Reduction** | **Usual Care** |
| 1 | Yes | Yes |
| 2 | Yes | No |
| 3 | Yes | No |
| 4 | Yes | Yes |
| 5 | Yes | Yes |

Table 1. Table to show which visits apply to which group.

| **Visit Number** | **Activity** | **Duration** | **Fasting**  **(if you opted in to the blood samples)** | **Location**  **(Transport costs will be reimbursed)** |
| --- | --- | --- | --- | --- |
| 1 | Baseline assessments (questionnaires, blood sample, physical assessments, wearing physical activity monitor) | 90 minutes | Yes | Your home, Russells Hall Hospital, or the University of Birmingham |
| 2 | Group or individual focus meeting and education about sedentary behaviour | 90 minutes | No | Your home (individual meeting), Russells Hall Hospital, or the University of Birmingham (group meeting) |
| 3 | Individual goal setting meeting | 60 – 90 minutes | No | Your home, Russells Hall Hospital, or the University of Birmingham |
| 4 | Pre-surgery measurements (questionnaires, blood sample, physical assessments, wearing physical activity monitor) | 90 minutes | Yes | Your home, Russells Hall Hospital, or the University of Birmingham |
| 5 | Post-surgery measurements (questionnaires, physical tests, wearing physical activity monitor) | 60 minutes | No | Your home, Russells Hall Hospital, or the University of Birmingham |

Table 2. Table to describe each of the study visits, what is to be done during each, and where they can take place.

**Are there any monetary costs to taking part?**

No, but you may choose to take a taxi or other form of public transport to meet the researcher for some of the visits. If you choose to do this, your travel costs will be covered by the study budget. Additionally, after fasting, if you choose to come to visits at either the School of Sport, Exercise and Rehabilitation Sciences at the University of Birmingham or Russells Hall Hospital, then breakfast will be provided from the study budget.

**Can I have more detail on what I will have to do?**

Over the course of the study, there are several procedures that we’d like you to do:

- ***Measurement of physical activity*** – we will attach a small physical activity monitor to your thigh using a sticky transparent dressing and ask you to wear it for 3 days (figure 1). The activity monitor has to be applied to the upper thigh, so we would ask you to remove some of your lower body clothing for a few minutes so that we can attach the monitor. If you are uncomfortable with this, the researcher can instruct you on how you can put on the monitor yourself. The monitors are very small (1.5x2.5 inches) and lightweight (less than 50 grams). You may bathe/ take a shower during this time (although if you wish to bathe we would ask you to remove the monitor while you are bathing). We will collect the monitor from you or provide you with a stamp-addressed envelope to return the monitor to us after 3 days. You will be asked to do this at visits 1, 4, and 5 in the study.

*
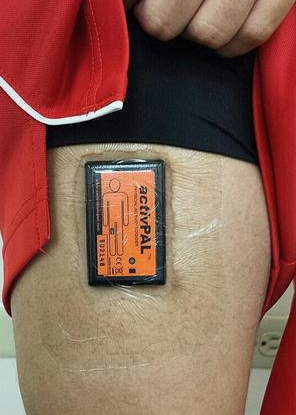
*

Figure 1 - Image depicting the ActivPal activity monitor attached to the thigh using a plastic adhesive.

- ***Questionnaires –*** throughout the study, we will ask you to complete questionnaires seven questionnaires. Some of them will be done two or three times throughout the study. These will relate to a number of aspects of the study and your health or wellbeing, including your quality of life, how your knee or hip affects your daily life, what you eat, your physical activity and sitting behaviour, and what you thought about the study.
- ***Physical measurements –*** physical measurements will include your weight, height, and waist-to-hip ratio, which is taken using a tape measure around your waist and hip.
- ***Test of functional ability (short physical performance battery) –*** This is a short series of tests of balance, walking speed (over a few feet) and chair rise. It will take about 10 minutes.
- ***Blood test (optional) –*** We will take small samples of blood from a vein (a total of 2 blood samples of 16ml each which is just a bit more than 2 tablespoons in total). We will take a sample at visit 1 and visit 4, and these may be taken either by a trained member of the study team or by a nurse. It will be required that you fast for 12 hours prior to these blood draws at these visits. These samples will be used to assess whether the study has had an effect on your risk for disease. In addition, a small tube of your blood will be sent to the University of Bologna in Italy as part of another study which is looking at how changes can occur in your genetic makeup over the course of your life, particularly as a result of studies like this one, and how these changes in your genes can be reflective of your age and the speed of ageing. This blood sample will be sent fully-anonymized and therefore the University of Bologna will not be able to identify you from the sample. The University of Bologna is a partner of the University of Birmingham and this study in an international collaboration known as the Physical Activity and Nutritional Influences in Ageing (PANINI) project and they have ethical approval to carry out this research. Results from this portion of the study may be published by the University of Bologna but you will not be identifiable in these publications. The blood samples are an optional part of the study and you can indicate on the consent form whether you’d like to take part in this.

**What are possible disadvantages or risks of taking part?**

The research team does not anticipate that you will be exposed to any risks that are much greater than your usual activities of daily living. Since how you change your sitting behaviour will be mostly determined by you in your goal-setting session with the researcher at the start of the study, the level of risk is likely to be very low.

**What are the possible benefits of taking part?**

Sitting for prolonged periods of time is associated with a greater risk of a number of diseases and a decreased ability to move around. By reducing your overall amount of sitting, or even by breaking up your sitting into smaller chunks, it is possible that your health and ability to move around will be improved. We hope this may also help you move about sooner and more capably after your surgery.

**Does taking part in the study affect the scheduling of my surgery in any way?**

No, taking part in the study will not affect your surgery date. Your healthcare providers will still make sure you get your surgery as soon as possible and the researchers running this study can not affect when your surgery takes place.

**How do I join the study?**

It is not required to return the contact agreement form to take part in the study. If you would like to participate in the study, you can contact the study team using either of these contact details:

Mr. Justin Aunger

Office: +44 (0)121 414 4125

Mobile: +44 (0)7341305544

[j.aunger@bham.ac.uk](mailto:j.aunger@bham.ac.uk)

Dr. Carolyn Greig

Tel: +44 (0)121 414 8743
[c.a.greig@bham.ac.uk](mailto:c.a.greig@bham.ac.uk)

Please leave a voicemail if the person you contacted doesn’t answer right away. If you prefer, you can also return the enclosed contact agreement form to the following address, after which you will be contacted by a member of the study team:

Dr. Carolyn Greig (INTEREST Study)
School of Sport, Exercise, and Rehabilitation Sciences
University of Birmingham
West Midlands
B15 2TT

**What if there is a problem?**

**Concerns and complaints:** If you have a concern about any aspect of this study, you should ask to speak with the researchers who will do their best to answer your questions. If you remain unhappy and wish to complain formally, you can do this by contacting Dr Sean Jennings, Research Support Group, University of Birmingham (0121 415 8011 or s.jennings@bham.ac.uk).

**Harm**: If something does go wrong and you are harmed during the research study, the University has in force a Public Liability Policy and/or Clinical Trials policy which provides cover for claims for "negligent harm" and the activities here are included within that coverage.

**Will my participation in the study be kept confidential?**

Any information that is collected about you during the course of the study will be kept strictly confidential, and any information that may leave the University will have all identifying information removed from it so that it cannot be traced back to you. Additionally, any data in publications which result from your taking part in the study will not contain identifying information.

The University of Birmingham is the sponsor for this study based in the United Kingdom. We will be using information from you in order to undertake this study and will act as the data controller for this study. This means that we are responsible for looking after your information and using it properly. The University of Birmingham will ensure identifiable information about you is kept securely for 10 years after the study has finished in line with the University’s Code of Practice for Research.

Your rights to access, change or move your information are limited, as we need to manage your information in specific ways in order for the research to be reliable and accurate. If you withdraw from the study, we will keep the information about you that we have already obtained. To safeguard your rights, we will use the minimum personally-identifiable information possible.

You can find out more about how we use your information by contacting the research team points of contact named towards the end of this information sheet or by contacting [researchgovernance@contacts.bham.ac.uk](mailto:researchgovernance@contacts.bham.ac.uk).

Your data may also be looked at by authorised representatives of the sponsor, the University of Birmingham, the NHS trust, or regulatory authorities in the case of trial-related monitoring, audits, and inspections.

The University of Birmingham will collect information from you for this research study in accordance with our instructions.

The University of Birmingham will use your name, contact details and date of birth to contact you about the research study, and make sure that relevant information about the study is recorded for your care, and to oversee the quality of the study. Individuals from the University of Birmingham and regulatory organisations may look at your medical and research records to check the accuracy of the research study. Russells Hall Hospital will pass these details to the University of Birmingham along with the information collected from you. The only people in the University of Birmingham who will have access to information that identifies you will be people who need to contact you to arrange study procedures or audit the data collection process. The people who analyse the information will not be able to identify you and will not be able to find out your name, date of birth or contact details.

The University of Birmingham will keep identifiable information about you from this study for 10 years after it ends.

**Involvement of the General Practitioner/Family Doctor (GP) and Surgical Consultant**

With your permission, we will inform your GP about your participation in this study. If any information arises during the study which could have an impact on your health then your GP or surgical consultant could be contacted to let them know.

**What will happen if I don’t carry on with the study?**

During the study, if you have a change of heart for any reason at any time then you can withdraw without having to give a reason. However, any data collected up until that point will be used.

**What happens after the study?**

After the study both groups will be provided with information about how to keep healthy while you age. If you’re in the intervention group, you will be able to keep any relevant study materials which can help you continue to work towards your goals.

**What will happen to the results of the research study?**

The results of this study will be published in medical journals, reports, and elsewhere. Your data will not be identifiable. Some of the data will be anonymously be added to the shared dataset within the PANINI project and shared with other European institutions, helping us to characterise older adult populations across Europe. This is optional and you can indicate on the consent form whether or not you are happy for us to do this.

**Who has reviewed the study?**

This study has been given a favourable ethical opinion for conduct in the NHS by the Solihull Research Ethics Committee.

**Who is organising or sponsoring the research?**

This study is sponsored by the University of Birmingham with RG number RG_17-169.

**Who can I talk to about taking part in research as a NHS patient?**

The Patient Advice and Liaison Service (PALS) can be contacted and they can provide independent advice on how to take part in research in a healthcare context.

**Further information and contact details:**

For additional information or other enquires please don’t hesitate to contact any of the research team by email or phone using the following information:

Mr. Justin Aunger

[j.aunger@bham.ac.uk](mailto:j.aunger@bham.ac.uk)

Office: +44 (0)121 414 4125

Mobile: +44 (0)7341305544

Elise Cooke & Heather Willis

Trauma & Orthopaedic Research Nurses

[heather.willis@nhs.net](mailto:heather.willis@nhs.net); [elise.cooke@nhs.net](mailto:elise.cooke@nhs.net)
Tel 01384 456111 ext 3730

Independent Sponsor Point of Contact:

Dr Sean Jennings

Head of Research Governance and Ethics

Researchgovernance@contacts.bham.ac.uk

Alternatively, you can send a letter to:

Dr. Carolyn Greig (INTEREST Study)
School of Sport, Exercise, and Rehabilitation Sciences
University of Birmingham
West Midlands
B15 2TT

[c.a.greig@bham.ac.uk](mailto:c.a.greig@bham.ac.uk)

Tel: +44 (0)121 414 8743

Thank you for your consideration of this study
